# Distinctive single-channel properties of α4β2-nicotinic acetylcholine receptor isoforms

**DOI:** 10.1371/journal.pone.0213143

**Published:** 2019-03-07

**Authors:** Maegan M. Weltzin, Andrew A. George, Ronald J. Lukas, Paul Whiteaker

**Affiliations:** Division of Neurobiology, Barrow Neurological Institute, Phoenix, Arizona, United States of America; Universidade de Sao Paulo Instituto de Quimica, BRAZIL

## Abstract

Central nervous system nicotinic acetylcholine receptors (nAChR) are predominantly of the α4β2 subtype. Two isoforms exist, with high or low agonist sensitivity (HS-(α4β2)_2_β2- and LS-(α4β2)_2_α4-nAChR). Both isoforms exhibit similar macroscopic potency and efficacy values at low acetylcholine (ACh) concentrations, mediated by a common pair of high-affinity α4(+)/(-)β2 subunit binding interfaces. However LS-(α4β2)_2_α4-nAChR also respond to higher concentrations of ACh, acting at a third α4(+)/(-)α4 subunit interface. To probe isoform functional differences further, HS- and LS-α4β2-nAChR were expressed in *Xenopus laevis* oocytes and single-channel responses were assessed using cell-attached patch-clamp. In the presence of a low ACh concentration, both isoforms produce low-bursting function. HS-(α4β2)_2_β2-nAChR exhibit a single conductance state, whereas LS-(α4β2)_2_α4-nAChR display two distinctive conductance states. A higher ACh concentration did not preferentially recruit either conductance state, but did result in increased LS-(α4β2)_2_α4-nAChR bursting and reduced closed times. Introduction of an α4(+)/(-)α4-interface loss-of-function α4W182A mutation abolished these changes, confirming this site’s role in mediating LS-(α4β2)_2_α4-nAChR responses. Small or large amplitude openings are highly-correlated within individual LS-(α4β2)_2_α4-nAChR bursts, suggesting that they arise from distinct intermediate states, each of which is stabilized by α4(+)/(-)α4 site ACh binding. These findings are consistent with α4(+)/(-)α4 subunit interface occupation resulting in allosteric potentiation of agonist actions at α4(+)/(-)β2 subunit interfaces, rather than independent induction of high conductance channel openings.

## Introduction

Nicotinic acetylcholine receptors (nAChR) are members of the ligand-gated ion channel superfamily of neurotransmitter receptors, with the first-to-be-identified muscle-type nAChR serving as a prototype [1]. In mammals functional pentameric nAChR subtypes with diverse pharmacological and biophysical properties, and distributions, are assembled from different combinations of nAChR subunits (α1—α7, α9, α10, β1—β4, γ, δ, ε). The endogenous neurotransmitter acetylcholine (ACh) activates function of all nAChR subtypes, and nicotine functions as an agonist of nAChR subtypes except those containing α9 subunits [2].

**Fig 1:**
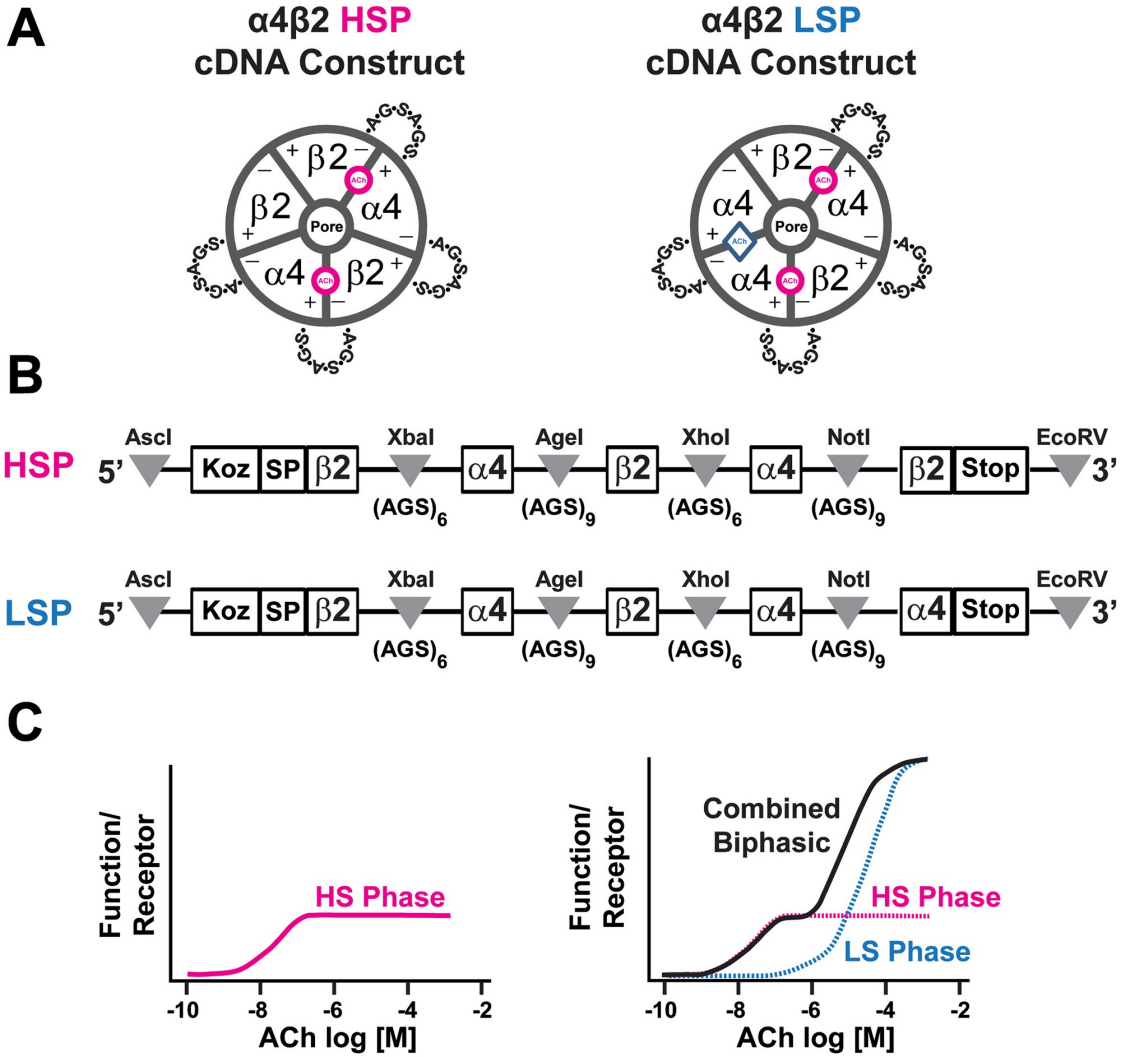
Schematic representations of high-sensitivity (α4β2)_2_β2- and low-sensitivity (α4β2)_2_α4-nAChR isoforms. **A)** Configuration of nAChR α4 and β2 protein subunits in each of the two isoforms. In this case, fully-concatenated pentameric isoforms are illustrated (HSP and LSP, respectively, with the short A-G-S peptide linkers that are used to enforce subunit associations shown). Note that both isoforms contain two canonical agonist binding sites (magenta circles) at the interfaces between the (+)-face of an α4 subunit and the (-)-face of a β2 subunit. The LS-isoform (α4β2)_2_α4-nAChR contains an additional agonist binding site (blue diamond) at the interface between the (+)- and (-)-faces of two α4 subunits. In receptors assembled from unlinked α4 and β2 nAChR subunits, mixed populations of the same two isoforms are typically formed. Each isoform exhibits the same ratios and associations of subunits as shown for the corresponding concatenated assembly, but A-G-S linkers are absent. It is also possible to produce essentially-pure populations of HS- or LS-isoform α4β2-nAChR by expressing strongly-biased ratios of unlinked subunits (see methods section for details). **B)** Illustration (from top to bottom) of cDNA constructs used to express concatenated HSP (α4β2)_2_β2- and LSP (α4β2)_2_α4-nAChR isoforms. Each construct is flanked with AscI and EcoRV restriction sites (5’ and 3’, respectively; indicated by gray triangles) for subcloning into high expression oocyte vectors. A Kozac sequence and the β2 signal peptide (SP) were retained only for the 1st position. Flanking each subunit position are unique restriction sites (indicated by gray triangles) used in concatemer design (for example, NotI and EcoRV were used in exchanging nAChR subunits at position 5). The two concatemers differ in composition only at position 5, containing either the β2 or α4 subunits in the HSP or LSP constructs, respectively. **C)** Illustrations of the whole-cell concentration response relationships of each α4β2-nAChR isoform. On the left, high-sensitivity isoform (α4β2)_2_β2-nAChR produce a single-phase response (shown in magenta, and resulting from ACh binding to the pair of high-affinity α4(+)/(-)β2 agonist binding sites. On the right, low-sensitivity isoform (α4β2)_2_α4-nAChR produce two response phases. One, again shown in magenta, is also produced by ACh engagement of the pair of α4(+)/(-)β2 agonist binding sites that is conserved across the two isoforms. This exhibits a similar EC_50_ value, and results in a similar amount of function per receptor, as is seen for the high-sensitivity isoform. Accordingly, it is named “HS-phase.” However, at higher ACh concentrations, a second and significantly-larger phase of function is observed. This emerges as a result of additional ACh binding to the low-sensitivity agonist binding pocket formed at the unique α4(+)/(-)α4 subunit interface. The combined response of (α4β2)_2_α4-nAChR has a characteristic biphasic appearance, and is dominated by the larger low-sensitivity phase. This has led to (α4β2)_2_α4-nAChR being named the low-sensitivity isoform.

nAChR containing α4 and β2 subunits (α4β2*-nAChR, where * indicates the known or possible presence of subunits in addition to those specified [3]) predominate within the mammalian central nervous system (CNS) [4]. Accordingly, α4β2*-nAChR contribute significantly to both normal and aberrant CNS function, with roles demonstrated in Alzheimer’s disease, learning, memory, epilepsy, mood, and nicotine self-administration and reward [5–20]. Both naturally- and heterologously-expressed α4β2-nAChR assemble as two functional isoforms [21–26], as illustrated in Fig 1A and 1B. Those receptors composed of two α4 and three β2 subunits produce a single phase of high-sensitivity (HS) whole-cell current (macroscopic) responses at low ACh concentrations and are therefore known as “HS (α4β2)_2_β2-nAChR” (see Fig 1C). The situation is more complex for the isoform containing three α4 and two β2 subunits (α4β2)_2_α4-nAChR), (see Fig 1C). At lower ACh concentrations, this isoform also has an HS response like that of HS (α4β2)_2_β2-nAChR. However, at higher ACh concentrations, a second, low-sensitivity (LS) phase of function is activated that generates much greater macroscopic current amplitude per receptor [27]. The resulting macroscopic ACh concentration/response relationship seen with (α4β2)_2_α4-nAChR is distinctively biphasic. The predominant LS phase of function prompted classification of (α4β2)_2_α4-nAChR as the LS α4β2-nAChR isoform.

Work by us and others has shown that two, canonical, orthosteric, high-affinity α4(+)/(-)β2 agonist-binding subunit interfaces are located in each of the HS and LS α4β2-nAChR isoforms. Here the (+) or (-) indicates the subunits providing, respectively, the so-called primary or complementary agonist binding components of the subunit interface. The critical distinction is that (α4β2)_2_α4-nAChR (i.e., LS isoform) also harbor a unique, lower-affinity, α4(+)/(-)α4 agonist binding site [27–30]. Agonist engagement of this LS isoform-specific site results in the previously-noted large increases in per-receptor function compared to HS α4β2-nAChR, which can only be activated by agonist binding at the common pair of α4(+)/(-)β2 sites [27]. The α4(+)/(-)α4 agonist binding site can therefore be considered to be functionally equivalent to a co-agonist or positive allosteric modulator site [27–29]. This phenomenon is responsible for the intrinsically biphasic ACh concentration-response profile of the LS (α4β2)_2_α4-nAChR isoform.

Our more-recent study demonstrated that the pair of α4(+)/(-)β2 agonist binding sites found in common in HS and LS isoform α4β2-nAChR contribute differently to macroscopic function between the two different isoforms [31]. This finding raised the question of whether correspondingly divergent responses at the individual receptor level might arise at low ACh concentrations, despite the apparently similar macroscopic activation characteristics of HS (α4β2)_2_β2- and LS (α4β2)_2_α4-nAChR below the concentration range at which the α4(+)/(-)α4 site of the LS isoform will become engaged by ACh.

In this study, single-channel, cell-attached electrophysiology was used to address this question, along with the related question of how LS (α4β2)_2_α4-nAChR function changes when the additional α4(+)/(-)α4 agonist binding site is engaged at a higher ACh concentration. We demonstrate that the HS and LS isoforms of α4β2-nAChR do indeed differ in their single-channel properties. Most notably, a single lower conductance state is associated with HS (α4β2)_2_β2-nAChR, whereas LS (α4β2)_2_α4-nAChR exhibit two distinct conductance states (neither of which matches that of HS (α4β2)_2_β2-nAChR). Intriguingly, increased function of the LS (α4β2)_2_α4-nAChR isoform at a higher ACh concentration (by engagement of the α4(+)/(-)α4 site) is associated with 1) a shift to shorter closed times, and 2) increased channel bursting, but 3) without preferential recruitment of either low- or high-conductance openings. Our findings shed new light on the complexity of, and differences between, the functional characteristics of the two α4β2-nAChR isoforms that appear so similar at the macroscopic level, even at an ACh concentration which acts only at the pair of ostensibly identical α4(+)/(-)β2 interfaces harbored within each isoform. These observations may also prove applicable to additional members of the nAChR family that host non-canonical agonist binding pockets [32, 33].

## Methods and materials

### Reagents

All reagents were purchased from Sigma (St. Louis, MO, USA) unless otherwise specified. Fresh solution stocks were made daily, diluted, and filtered as required.

### DNA constructs and cRNA synthesis

#### Heterologous expression of unlinked human α4 and β2 nAChR subunits

As previously described [20], full-length cDNA for human wild-type α4 (NCBI Reference Sequence: NM_000744.5) and β2 (NCBI Reference Sequence: NM_000748.2) subunits were synthesized (Life Technologies, Grand Island, NY, USA). Preparation of cRNA from these subunits, ligated into the pCI mammalian expression vector (Promega Madison, WI, USA), was also as previously described [20]. cRNA purity was confirmed on a 1% agarose gel, and final products were sub-aliquoted and stored at -80°C.

#### Heterologous expression of concatenated human α4 and β2 nAChR subunits

HS or LS α4β2-nAChR tethered pentameric concatemers (human subunits were linked with AGS repeats enforcing β2-α4-β2-α4-β2 [HSP; HS tethered pentamer] or β2-α4-β2-α4-α4 [LSP; LS tethered pentamer] subunit arrangements, respectively; see Fig 1B) were the same as those previously described [27, 31]. Using concatenated receptors, we were able to ensure expression of pure populations of either HS or LS α4β2-nAChR, allowing us to cross-verify our conclusions using recordings made with nAChR expressed from unlinked subunits. HSP and LSP cDNA and cRNA were synthesized similarly to those of unlinked subunits, as detailed in [27, 31].

To further confirm the α4(+)/(-)α4 ACh binding site’s contribution to distinctive elements of LS α4β2-nAChR function, we introduced a tryptophan (W) to alanine (A) mutation at amino acid 182 of only the α4 subunit at LSP position 5 (LSP-α4p5W182A; subunit order β2-α4-β2-α4-α4[W182A]). Residue W182 is located in the B-loop, on the α4(+) face of the α4(+)/(-)α4 agonist binding site that is uniquely contained in the LSP α4β2-nAChR isoform. The LSP-α4p5W182A construct encodes a receptor that exhibits dramatically-reduced, low sensitivity agonist activation [27]. cDNA synthesis and cRNA transcription followed methods described for the unmodified LSP concatemer construct, and in [27].

### Oocyte isolation and cRNA microinjections

*Xenopus laevis* harvested and de-folliculated stage V oocytes were purchased from EcoCyte Bioscience (Austin, TX, USA). cRNA microinjection and oocyte incubation conditions precisely followed methods described in previous studies [20, 27]. Expression of either HS or LS α4β2-nAChR in *Xenopus* oocytes from un-linked subunits was achieved by injection of different cRNA subunit ratios (1 ng of α4: 10 ng of β2 for HS (α4β2)_2_β2-nAChR or 30 ng of α4: 1 ng of β2 for LS (α4β2)_2_α4-nAChR, respectively). Oocytes expressing HS or LS isoforms assembled from unlinked subunits were recorded from at 3–6 days post cRNA injection. These conditions were chosen on the basis of our previous work, which demonstrated that these subunit injection ratios produce essentially uniform populations of the desired α4β2-nAChR isoforms within this timeframe [20].

For concatenated nAChR constructs, 20 ng of HSP, LSP or LSP-α4p5W182A cRNA were injected into oocytes. For these constructs, a longer incubation time was needed between injection and recording (5—10d), because expression of functional pentameric α4β2-nAChR at the cell surface is slower to emerge from concatenated constructs than is the case for nAChR assembled from unlinked subunits.

### Single-channel patch-clamp electrophysiological recordings

Single-channel electrophysiological recordings from *Xenopus* oocytes expressing α4β2-nAChR were obtained under conditions similar to those used previously [34]. Oocytes were manually stripped of the vitelline membrane using sharp forceps under a dissecting microscope (magnification = 20x total magnification) and transferred to a recording chamber containing oocyte Ringer’s solution (OR2; 92.5 mM NaCl, 2.5 mM KCl, 1 mM MgCl_2_∙6H_2_O, 1 mM CaCl_2_∙2H_2_O, and 5 mM HEPES; pH 7.5). Atropine sulfate (1.5 μM) was added to all recording and bath solutions to block any potential muscarinic responses. Patches were formed in cell-attached mode, at 22°C. Recording patch pipettes were pulled from thick-walled (2 mm outer diameter, 1.12 mm inner diameter) borosilicate glass capillary tubes (World Precision Instruments, Inc., Sarasota, FL, USA). Electrodes were fire polished using a World Precision Instruments microforge to a final resistance of 15–20 mΩ. Recordings were obtained using an Axopatch 200B amplifier (Molecular Devices, Sunnyvale, CA, USA), filtered on-line at 5 kHz, digitized at 50 kHz using an Axon Digidata 1550 (Molecular Devices), and stored on a personal computer for later analysis. Inward single channel α4β2-nAChR currents were elicited with ACh (OR2 + ACh within the patch electrode), and measured at +70 mV holding potential (corresponding to a transmembrane potential of approximately -100 mV). To maximize recording quality, patches with seal resistance < 8 GΩ were immediately discarded. All patches were tested for the presence of endogenous stretch channels by applying negative pressure to each patch. Data were discarded from any patches in which mechanosensitive events were observed. Under the preceding conditions, we observed no channel openings from oocytes expressing any of the α4β2-nAChR constructs in the absence of ACh.

Choices of ACh concentrations were dictated by the need to collect sufficient numbers of events to analyze, while avoiding potential overlap of unitary events and/or channel block at very high ACh concentrations. Concentrations below the HS ACh EC_50_ value produced few events, making subsequent analysis difficult. Accordingly, ACh concentrations were chosen as corresponding to isoform-specific EC_50_ values, determined in previous two-electrode voltage-clamp recordings [20, 27, 31]. Specifically, unlinked subunit HS or concatenated HSP α4β2-nAChR single channel responses were stimulated using 1.3 μM ACh (low concentration). As previously stated, macroscopic currents mediated by LS α4β2-nAChR have biphasic ACh concentration/response profiles [20, 27–29, 31]. Accordingly, we studied unlinked subunit LS and concatenated LSP α4β2-nAChR (including LSP-α4p5W182A) in the presence of 0.7 μM ACh (HS phase EC_50_; low concentration) to stimulate just HS responses or in the presence of 30 μM ACh (LS phase EC_50_; high concentration) to activate both HS and LS responses.

### Single-channel patch-clamp electrophysiology data analysis

Single channel response data recordings were filtered off-line at 1 kHz and analyzed using the model-based-analysis program QuB (www.qub.buffalo.edu); [35]. Single channel records were analyzed using the segmental K-means (SKM) idealization method to measure unitary amplitudes, event durations, and open probabilities. The maximum interval likelihood (MIL) feature was used to determine open and closed dwell time distributions [36, 37]. The number of states (used to generate open and closed dwell time histograms) was determined by adding additional open or closed states to the receptor model. An optimal number of states were determined once the log likelihood (LL) algorithm failed to improve the model by > 10 LL units. Stability plots were generated for all HS α4β2-nAChR, and were used to examine systematic changes over time in amplitudes, open, and closed dwell time distributions. More-extended recording periods typically resulted in longer measured durations of closed states, suggesting a receptor run-down phenomenon. For this reason, only data collected during the first 60s of recordings from HS α4β2-nAChR were analyzed (since stability plots showed that changes occurred after this cut-off time). Function within patches containing LS α4β2-nAChR was shown by the same stability plot approach to be more stable, allowing data from the first 120s to be used. This relatively-rapid rundown phenomenon for both isoforms is similar to that recently reported for α4β2-nAChR [38].

All constructs were tested using expression from at least three separately-synthesized batches of cRNA, and at least three separate batches of oocytes. In order to ensure that, for each construct, individual patches were comparable in terms of numbers of nAChR present and functional status, each patch was examined for consistency as measured by the number of events per second. A two standard deviations (SD) outlier test was applied, and patches that had > 2 SD of the mean more or fewer events per second were excluded from further analysis. The rationale was that *hyper*-eventful patches likely contained atypically large numbers of nAChR, and *hypo*-eventful patches likely contained desensitized, inactivated, or run-down nAChR. Subsequent to the implementation of this exclusion criterion, each experimental group contained 5–10 single-channel recordings that were used for further data analysis.

Single-channel function of both α4β2-nAChR isoforms occurred as a mixture of single openings and (less-frequent) short bursts of channel openings, interspersed with longer-duration closed dwell periods. In addition to studying properties of all individual openings, we chose to analyze functional parameters associated with bursts. This enhances the likelihood that adjacent openings arise from the same receptor [39, 40]. To this end, bursts were defined as series of two or more openings separated by closures shorter than a minimum interburst closed duration, or T_crit_, chosen to minimize the number of misclassified closed events [41, 42]. For all constructs tested, T_crit_ was calculated using QuB software. Very few bursts containing overlapping currents were observed. Since these represented simultaneously active channels, they were discarded from analysis. In patches containing LS α4β2-nAChR, bursts fell into two populations, with small and large amplitudes. Almost no bursts were recorded that contained a transition between small and large amplitude openings–any such bursts were also discarded from analysis. Differentiation between LS isoform bursts containing small or large amplitude openings was achieved using the QuB X-means algorithm to separate the two populations [43], which were then analyzed separately. QuB was also used to quantify burst properties such as the proportion of openings found within bursts, numbers of openings within bursts, and open probability within a burst (P_open_). Exported QuB data were used to determine burst duration values using exponential log probability histograms generated by Clampfit 10.4.1.4 software (Molecular Devices).

### Single-channel conductance measurements

In addition to amplitude measurements at a single transmembrane potential (-100 mV; described previously), single-channel slope conductance values were estimated in separate experiments. In these studies, the linear fit of the mean current amplitude was calculated, recorded across a range of transmembrane potentials (-70 to -140 mV, in steps of 10 mV). Amplitude histograms were generated as described in the preceding section, for each holding potential, in each patch.

### Statistical analysis

Results are presented as mean ± S.E.M., except for error estimates associated with Gaussian or exponential distributions (which are described as histograms with the best fit value ± standard error of the mean [S.E.M.]). Measured single-channel functional parameters were statistically analyzed using Prism 5.03 Software (La Jolla, CA, USA), and the number of patches was used as N. Two-tailed unpaired student’s t-tests were used to compare pairs of groups. One-way analysis of variance (ANOVA) and Tukey’s multiple comparison tests were used to evaluate the means of three or more groups and differences between them.

## Results

Data collected from nAChR expressed using unlinked subunits are depicted in gray (HS (α4β2)_2_β2-nAChR) or green (LS (α4β2)_2_α4-nAChR). Those collected from nAChR expressed using concatenated subunits are shown in magenta (HSP isoform) or cyan (LSP isoform). Primary data collected from concatenated LSP α4β2-nAChR containing mutant subunits are illustrated in red.

### α4β2-nAChR unitary amplitudes and conductances are isoform specifi*c*

**Fig 2:**
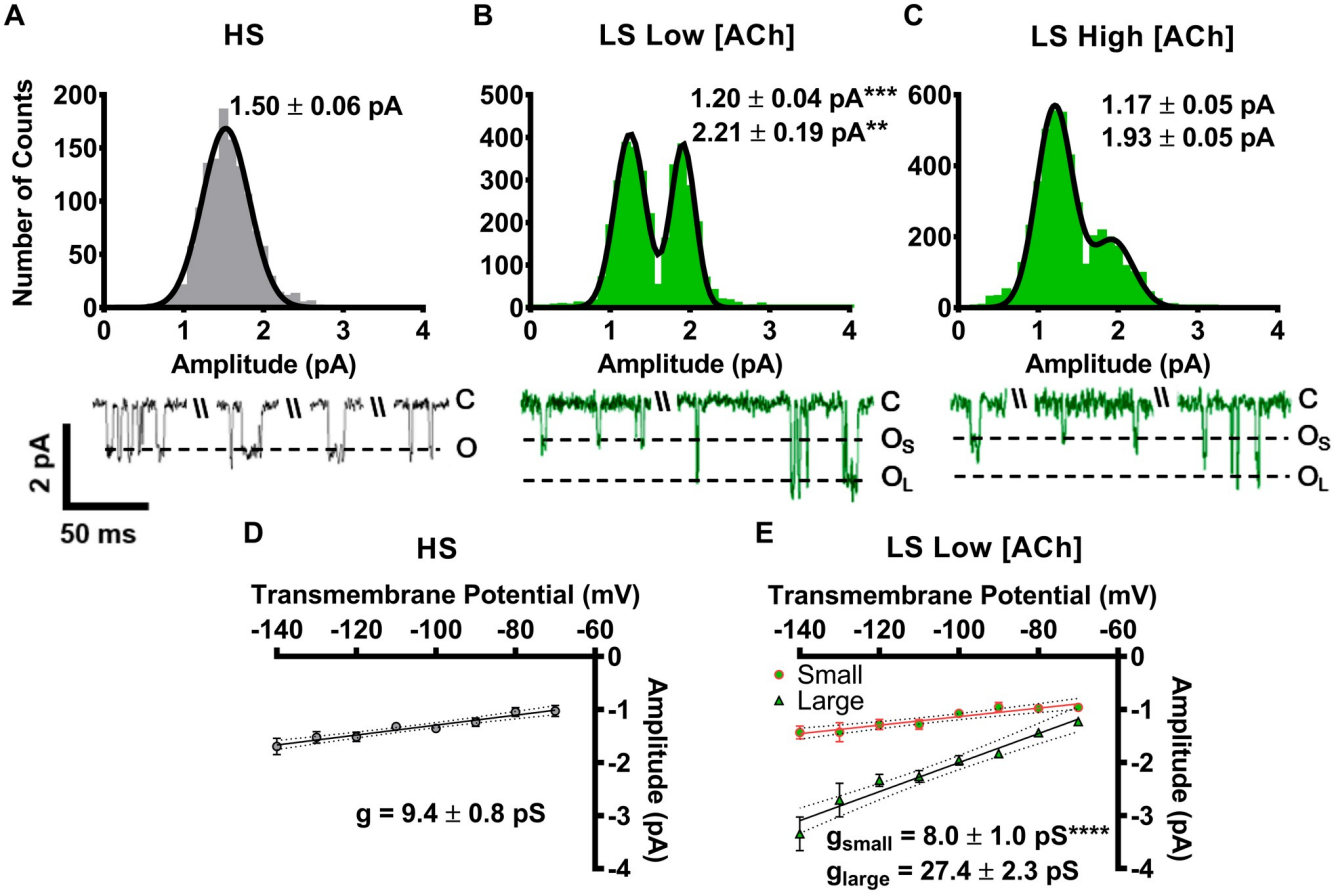
Unitary amplitudes and conductances associated with human HS (α4β2)_2_β2-nAChR and LS (α4β2)_2_α4-nAChR expressed in *X*. *laevis* oocytes from unlinked subunits. **(A)** HS (α4β2)_2_β2-nAChR (grey bars) open amplitudes were found to fall into a single population. **(B)** LS (α4β2)_2_α4-nAChR (green bars) single channel openings were recorded in the presence of a low ACh concentration (0.7 μM). Under these conditions, openings exhibited two characteristic amplitudes (small and large). Both the small and large amplitudes were significantly different than that associated with opening of HS (α4β2)_2_β2-nAChR (F_2,17_ = 12.38, One-way ANOVA; Tukey’s multiple comparison test indicates ** P < 0.01, and *** P < 0.001). **(C)** LS (α4β2)_2_α4-nAChR stimulated with a high ACh concentration (30 μM) also displayed two distinct amplitudes, which were statistically indistinguishable from those recorded in panel (B), using a low ACh concentration (two-tailed unpaired Student’s t-test were applied to each amplitude class; df = 11–13 and P > 0.05 in each case). Amplitude histograms are all of the events for each construct and ACh concentration collected from individual single-channel patch recordings. Example traces are shown below panels (A), (B), and (C), exhibiting a typical mixture of individual openings and short bursts of activity, interspersed with longer periods of inactivity. **(D)** Single channel slope conductance was calculated for openings of HS (α4β2)_2_β2-nAChR by calculating the mean single-channel amplitude across several transmembrane voltages. **(E)** The single-channel slope conductances associated with small and large openings of LS (α4β2)_2_α4-nAChR (g_small_ and g_large_, respectively) were also significantly different from each other (**** P < 0.0001; two-tailed unpaired Student’s t-test, df = 6). The conductance value g_small_ was similar to that associated with openings of the HS isoform. Values are given as mean ± S.E.M, and were collected from 5–9 patches across a minimum of three separate experiments. All recordings were performed 3—6d following cRNA injection.

We began by measuring amplitudes of all single channel events associated with the two α4β2-nAChR isoforms (*i*.*e*., across both isolated single openings and openings within bursts). These initial measurements were taken using a transmembrane potential of -100 mV. As shown in Fig 2, individual openings of both α4β2-nAChR isoforms (when expressed from unlinked subunits) were observed to occur both in isolation and as short bursts. Longer-duration closed dwell periods were interspersed between both patterns of openings, for both isoforms. For HS (α4β2)_2_β2-nAChR, only a single amplitude state (1.50 ± 0.06 pA; Fig 2A) was observed. In contrast LS (α4β2)_2_α4-nAChR displayed two open amplitudes, termed small (O_s_) and large (O_L_), at both the low ACh concentration (small 1.20 ± 0.04 pA; large 2.21 ± 0.19 pA; Fig 2B) and at the high ACh concentration (small 1.17 ± 0.05 pA; large 1.93 ± 0.05 pA; Fig 2C). The amplitudes of the small and large LS (α4β2)_2_α4-nAChR isoform openings did not significantly change between low and high ACh concentrations. This demonstrated that channel block was not a significant factor even at the higher agonist concentration (as previously observed [44], open channel block by agonists manifests as fast “flicker” of open currents as the agonist rapidly and sequentially enters and leaves the ion-channel. When data are filtered in order to remove high-frequency noise, the effect is to average the apparent size of openings across the peaks and troughs of the flicker, producing a reduction of the observed open amplitude(s)). Although the smaller unitary amplitude measured for LS (α4β2)_2_α4-nAChR was suggestively similar to that associated with HS (α4β2)_2_β2-nAChR openings, statistical analysis indicated that it was significantly different (Fig 2B).

Slope conductances of the HS (α4β2)_2_β2 and LS (α4β2)_2_α4 isoforms also were determined by measuring unitary amplitudes across a range of transmembrane voltages. HS (α4β2)_2_β2 had a slope conductance of 9.4 ± 0.8 pS (Fig 2D), whereas small and large amplitude events produced by LS (α4β2)_2_α4 isoform were associated with slope conductances of 8.0 ± 1.0 pS and 27.4 ± 2.3 pS, respectively (Fig 2E). In this case, the LS (α4β2)_2_α4-nAChR small-opening conductance was statistically indistinguishable from that of the single conductance associated with HS (α4β2)_2_β2-nAChR (Fig 2D *vs*. 2E). However, larger-amplitude events produced by LS (α4β2)_2_α4-nAChR were associated with a significantly higher conductance state compared to the HS isoform conductance and to LS isoform small-opening events.

The results just described appeared to indicate that LS (α4β2)_2_α4-nAChR produce two different classes of single-channel events, associated with two different conductance states. However, it is possible in principle that use of even a highly biased ratio of unlinked subunits (30:1 α4:β2) could have produced a mixed population of (α4β2)_2_α4- and (α4β2)_2_β2-nAChR isoforms, rather than the essentially-pure LS (α4β2)_2_α4-nAChR population intended. To determine if this was the case, oocytes were injected with concatenated (α4β2)_2_β2- or (α4β2)_2_α4-nAChR pentameric constructs (HSP and LSP, respectively). These are designed to ensure expression of fully defined HS or LS pentameric α4β2-nAChR isoforms.

**Fig 3:**
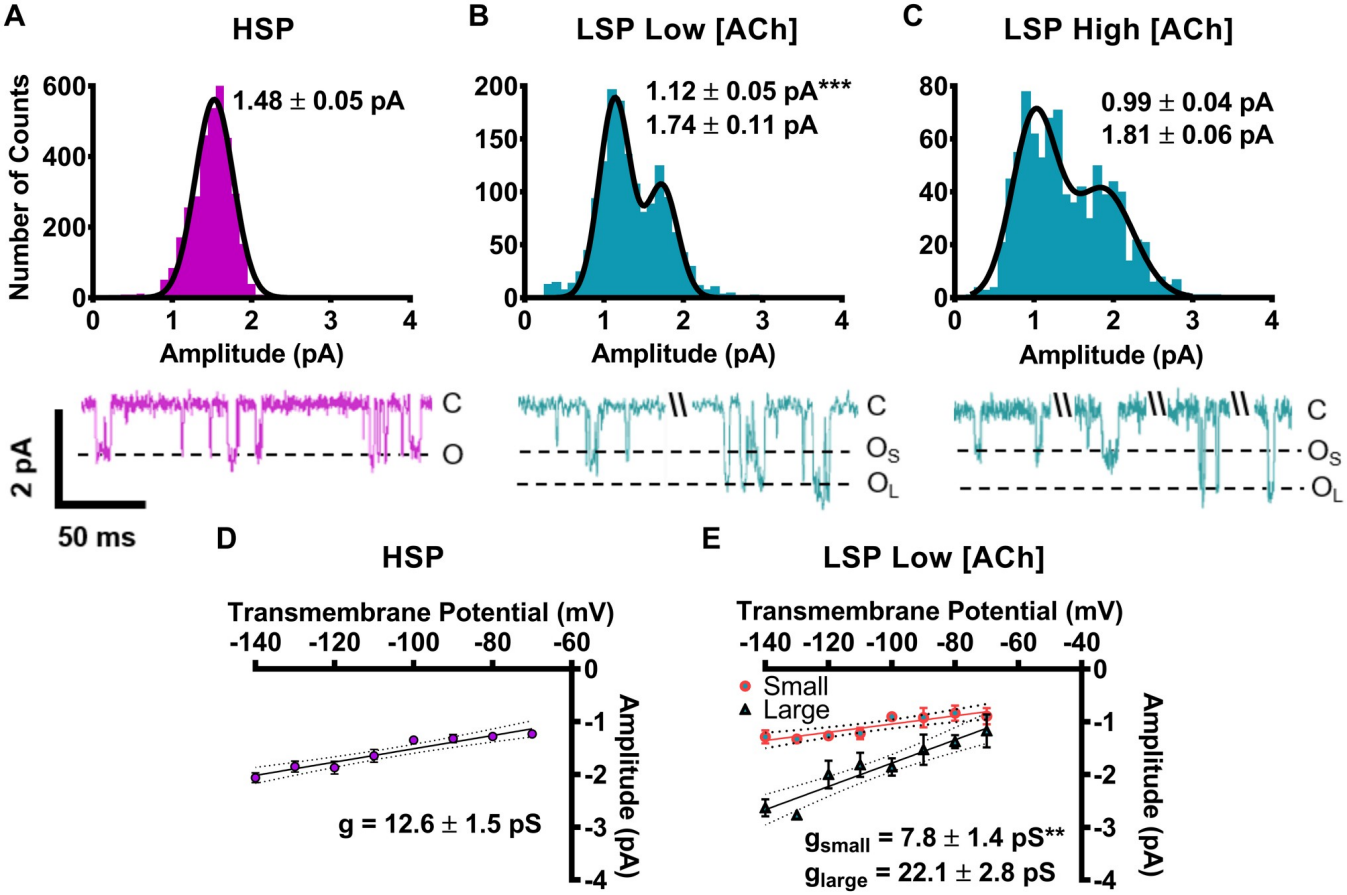
Unitary amplitudes and conductances associated with human HSP (α4β2)_2_β2-nAChR and LSP (α4β2)_2_α4-nAChR expressed in *X*. *laevis* oocytes from pentameric, fully-concatenated constructs (HSP and LSP, respectively). All statistical comparisons for this figure were performed using two-tailed unpaired Student’s t-tests, df = 8–13, significant differences were noted at P < 0.05. **(A)** HSP (α4β2)_2_β2-nAChR (magenta bars) stimulated with 1.3 μM ACh produced single-channel openings with a single characteristic amplitude which was indistinguishable from that measured in Fig 2A, for the same isoform expressed using unlinked subunits. **(B)** LSP (α4β2)_2_α4-nAChR (cyan bars) single-channel openings were recorded in the presence of a low ACh concentration (0.7 μM). These openings fell into two amplitude classes (small and large), associated with values indistinguishable from those determined in Fig 2B, for the same isoform expressed using unlinked subunits. **(C)** LSP (α4β2)_2_α4-nAChR were stimulated with a high ACh concentration (30 μM). Openings displayed two distinct amplitudes, which were statistically indistinguishable from those recorded in panel (B), using a low ACh concentration. Amplitude histograms are all of the events for each construct and ACh concentration from individual single-channel patch recordings. As in Fig 2, example traces are shown below panels (A), (B), and (C), which display a mixture of individual openings and short bursts of activity, interspersed with longer periods of inactivity. **(D)** Single-channel slope conductance was calculated for openings of HSP (α4β2)_2_β2-nAChR by calculating the mean single-channel amplitude across several transmembrane voltages. The value obtained was not statistically different to that recorded from (α4β2)_2_β2-nAChR expressed from unlinked subunits. **(E)** The single-channel slope conductances associated with small and large openings of LSP (α4β2)_2_α4-nAChR (g_small_ and g_large_, respectively) were also determined. The conductance value g_small_ was significantly lower than that associated with openings of the HSP isoform (* P < 0.05) and significantly lower than that associated with the large openings of the LSP isoform (** P <0.01). Conductances of small and large amplitude events expressed using the concatemer approach did not differ from events produced by the loose subunit technique. Values are given as mean ± S.E.M, and were collected from 5–6 patches across a minimum of three separate experiments. All recordings were performed 3—6d following cRNA injection.

As shown in Fig 3, outcomes when using fully-linked, concatemeric constructs were essentially identical to those produced using biased ratios of unlinked subunits. HSP α4β2-nAChR exhibited a single amplitude state of the same size as previously noted HS isoform, loose subunit receptors (Figs 3A *vs*. 2A). Most importantly, the LSP-isoform at both ACh concentrations had two unitary amplitudes; small and large, again similar in size irrespective of the ACh concentration and expression technique (Figs 3B and 3C *vs*. 2B and 2C). In a further point of similarity between the biased-ratio loose-subunit and concatenated-subunit approaches, the LSP isoform small amplitude size at either ACh concentration was significantly smaller than the sole amplitude associated with HSP isoform openings (Fig 3A *vs*. 3B). In a final complement to the initial unlinked-subunit experiments, we also determined the channel conductances associated with the HSP- and LSP-isoform α4β2-nAChR populations (Fig 3D and 3E). The distinct channel conductances associated with the two isoforms remained unchanged, whether expressed from linked or unlinked subunits (Figs 3D and 3E *vs*. 2D and 2E).

### Closed dwell time distributions distinguish α4β2-nAChR HS and LS isoforms, when expressed from unlinked subunits

To characterize single channel closed times, we next performed analysis of closed dwell times between all open events. The briefest component (τ_1_) is considered to occur within bursts, while longer components (τ_2_, *etc*.) correspond to closings that occur between independent openings, bursts, or both [45].

**Fig 4:**
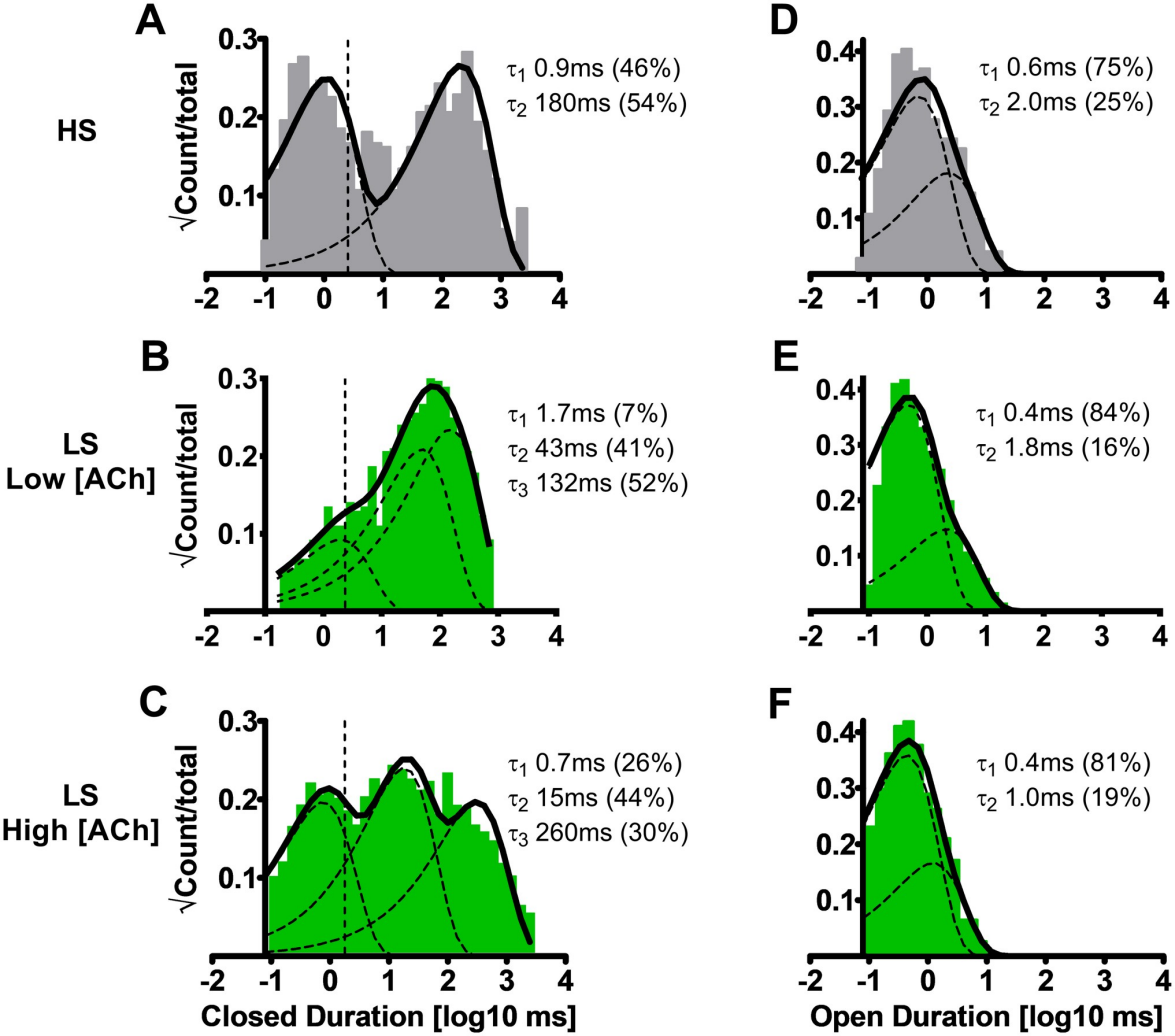
Closed and open dwell durations associated with human HS or LS α4β2-nAChR isoforms expressed in *X*. *laevis* oocytes from unlinked subunits. HS (α4β2)_2_β2-nAChR stimulated with ACh (1.3 μM). Closed durations between openings were best described with a pair of time constants **(A)**, as were open durations **(D)**. When LS (α4β2)_2_α4-nAChR were stimulated at a low ACh concentration (0.7 μM), closed durations between openings were best described with three time constants **(B)**, while open durations were best fit using two time constants **(E)**. The numbers of time constants required to best fit closed **(C)** and open **(F)** time distributions did not change as LS isoform nAChR were stimulated at a higher ACh concentration (30 μM), but the closed dwell times durations did shorten significantly (see Table 1). The open duration constants did show a trend towards shortening as the ACh concentration was increased, but this did not reach significance in this preliminary analysis (which did not discriminate between open events within and outside of bursts). Closed and open dwell duration histograms are representative examples collected from individual single-channel patch recordings. Individual τ values and percentage of total events corresponding to each closed and open durations from an example patch recording have been inserted into each panel to facilitate interpretation. Data were collected from 6–9 individual patches, across at least three separate experiments. Calculated parameters are summarized in Table 1, together with the statistical analyses applied.

The closed-time kinetics of HS (α4β2)_2_β2-nAChR, which were expressed using loose subunits, were best fit with two closed durations (Fig 4A; Table 1). However, LS (α4β2)_2_α4-nAChR expressed using unlinked subunits had closed dwell time histograms that were best fit with three exponential components. This was true whether LS (α4β2)_2_α4-nAChR were stimulated using either the low or high ACh concentrations (Fig 4B and 4C, respectively; Table 1).

**Table 1:** Closed and open dwell duration parameters for human high sensitivity (HS) and low sensitivity (LS) α4β2-nAChR isoforms expressed using unlinked subunits.

Isoform	Number of Patches	Tcrit ± SEM (ms)	Closed Durations ± SEM (ms) *(% ± SEM)*			Open Durations ± SEM (ms) (% ± SEM)	
			τ_1_	τ_2_	τ_3_	τ_1_	τ_2_
**HS (α4β2)**_**2**_**β2-nAChR (unlinked subunits), *low [ACh] (1*.*3 μM)***							
(α4β2)_2_β2	9	2.7 ± 0.4	0.9 ± 0.2 *(41 ± 7%)*	500 ± 100 *(63 ± 8%)*	Absent	0.7 ± 0.2 *(69 ± 9%)*	2.1 ± 0.3 ^Ϯ^ *(31 ± 9%)*
**LS (α4β2)**_**2**_**α4-nAChR (unlinked subunits), *low [ACh] (0*.*7 μM)***							
(α4β2)_2_α4	6	3.1 ± 0.5	1.3 ± 0.3 *(11 ± 2%)*	120 ± 40 *(38 ± 7%)*	700 ± 200 *(40 ± 10%)*	0.5 ± 0.1 *(80 ± 7%)*	2.4 ± 0.5^Ϯ^ *(20 ± 7%*^Ϯ^*)*
**LS (α4β2)**_**2**_**α4-nAChR (unlinked subunits), *high [ACh] (30 μM)***							
(α4β2)_2_α4	7	1.6 ± 0.2	0.66 ± 0.08* (25 ± 3%*)	28 ± 6* (35 ± 5%)	290 ± 80 *(38 ± 5%)*	0.37 ± 0.02 *(70 ± 10%)*	1.4 ± 0.2^Ϯ^ *(30 ± 10%*^Ϯ^*)*

HS isoform single channel events were elicited at a low ACh concentration, corresponding to the macroscopic EC_50_ value at this isoform (1.3 μM). LS isoform activity was stimulated at two different ACh concentrations (0.7 μM; low, or 30 μM; high), corresponding to macroscopic EC_50_ values for the HS and LS phases of function, respectively. In this initial analysis, all events were included whether or not they were associated with bursts of openings. Data represent τ mean ± SEM of parameters derived from individual patches during three or more individual experiments, with the number of patches noted in each case. All comparisons were performed using Student’s two-tailed, unpaired, t-tests (df = 11–13, significance noted when P < 0.05).

For HS (α4β2)_2_β2-nAChR, closed durations are best described with two components. Percentages of total events corresponding to each time constant, τ_1_ and τ_2_, are shown in parentheses under their associated time constants. This convention is followed for all other open or closed duration constants shown throughout this table. Individual openings produced by HS α4β2-nAChR all have the same mean amplitude (see Fig 2), but exhibit two statistically-distinct open durations (^Ϯ^).

For LS (α4β2)_2_α4-nAChR, closed durations are best described with three components. Significant changes in single-channel closed dwell durations are evident between LS-isoform α4β2-nAChR stimulated at low and high ACh concentrations for τ_1_ and τ_2_ (*). The proportion of LS isoform closed events classified within τ_1_ also significantly increase at the high ACh concentration compared to that seen for the low ACh concentration (*), although proportions of events falling into τ_2_ and τ_3_ are not significantly altered. Individual open events for LS isoform α4β2-nAChR are best fit with two significantly-different components (^Ϯ^). A larger proportion of total LS isoform events occur during τ_1_ compared to τ_2_ at either ACh concentration (^Ϯ^). Different ACh concentrations do not produce significant changes in open durations or proportions of events assigned to the two open-time components measured for LS α4β2-nAChR.

At the lower ACh concentration, LS (α4β2)_2_α4-nAChR closed τ_1_ and τ_3_ values were similar to those for HS (α4β2)_2_β2-nAChR closed τ_1_ and τ_2_ values (Fig 4A and 4B; Table 1). However, the LS (α4β2)_2_α4-nAChR intermediate closed time constant (τ_2_) appeared to be unique. Moving to the higher ACh concentration is thought to engage the additional α4(+)/(-)α4 agonist binding site found uniquely in LS (α4β2)_2_α4 [27–29]. At the tested high ACh concentration (30 μM), closed dwell times of the LS isoform were significantly shortened when compared to LS isoform single-channel closed times recorded at low (0.7 μM) ACh concentrations (for τ_1_ and τ_2_, with a trend of τ_3_ also being reduced; Fig 4B and 4C; Table 1). A significant increase in the percentage of events found in the fastest τ_1_ category (i.e., within-burst closed times) was also observed as the ACh concentration was raised (Table 1).

### Open durations are similar between the HS and LS isoforms when expressed from unlinked subunits

Open time distributions were also determined for HS (α4β2)_2_β2- or LS (α4β2)_2_α4-nAChR expressed from unlinked subunits. This initial analysis was performed for all individual openings, regardless of whether they fell within bursts or not. All open time histograms, regardless of ACh concentration or isoform, were best fit with two components (Fig 4D–4F;Table 1). At the low ACh concentration, events arising from both α4β2-nAChR isoforms exhibited very similar mean open times and distributions between τ_1_ and τ_2_. When the ACh concentration was increased, there was a trend to decreased open-event durations of LS (α4β2)_2_α4-nAChR, but this did not reach significance (Fig 4E and 4F; Table 1).

### Concatenated HSP and LSP α4β2-nAChR closed- and open-durations mirror those of HS (α4β2)_2_β2- and LS (α4β2)_2_α4-nAChR assembled from unlinked subunits.

**Fig 5:**
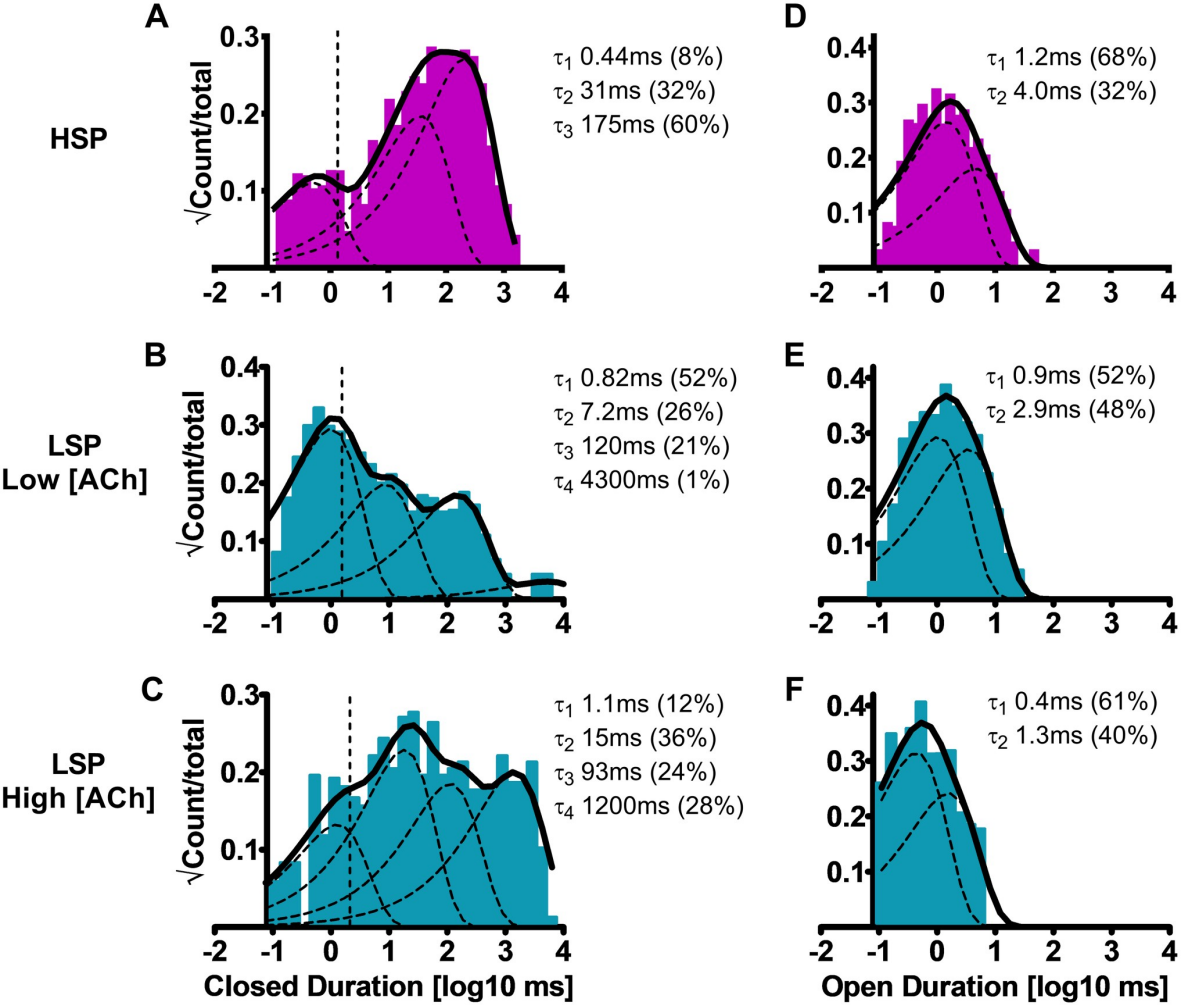
Closed and open durations associated with human HSP or LSP α4β2-nAChR isoforms expressed in *X*. *laevis* oocytes from pentameric, fully-concatenated constructs. HSP (α4β2)_2_β2-nAChR were stimulated with ACh (1.3 μM). Closed dwell durations between openings were best described with three time constants **(A)**, while open durations were best described with a pair of time constants **(D)**. When LSP (α4β2)_2_α4-nAChR were stimulated with a low ACh concentration (0.7 μM), closed durations between openings were best described with four time constants **(B)**, while individual-event open times were best fit using three time constants **(E)**. As was seen for unlinked subunits, increasing the ACh concentration to 30 μM did not change the number of time constants required to best fit closed **(C)** and open **(F)** duration distributions, but shortening of closed dwell durations was observed (see Table 2). Open dwell duration values did significantly shorten at the higher ACh concentration (Table 2). Closed and open dwell duration histograms are representative examples resulting from analysis of individual single-channel patch recordings. Individual τ values and percentage of total events corresponding to each closed and open duration from an example patch recording have been inserted into each panel to facilitate interpretation. Data were collected from 5–8 individual patches, across at least three separate experiments. Calculated parameters are summarized in Table 2, together with the statistical analyses applied.

We also measured closed- and open-state durations for receptors assembled from fixed-stoichiometry and arrangement HSP and LSP pentameric, concatenated α4β2-nAChR constructs, in the same way as just described for α4β2-nAChR isoforms assembled from unlinked subunits. Compared to the two closed states observed for HS (α4β2)_2_β2-nAChR expressed from unlinked subunits, HSP (α4β2)_2_β2-nAChR closed dwell time data were best fit with an additional closed state (three states; Table 2; Figs 5A *vs*. 4A). Similarly, four closed states observed for LSP (α4β2)_2_α4-nAChR contrasted with the three closed dwell times for LS (α4β2)_2_α4-nAChR assembled from unlinked subunits (Table 2; Figs 5B and 5C *vs*. 4B and 4C), at both the low and high ACh concentrations tested. However, in a point of similarity between nAChR expressed from linked and unlinked subunits, LSP α4β2-nAChR closed dwell times again showed a trend toward shorter durations at the higher ACh concentration, although this did not reach significance for any of the four identified components (Table 2).

**Table 2:** Closed and open dwell duration parameters for human HS and LS α4β2-nAChR isoforms expressed using fully-pentameric concatemeric constructs (HSP and LSP, respectively).

Isoform	Number of Patches	T_crit_ ± SEM (ms)	Closed Durations ± SEM (ms) *(% ± SEM)*				Open Durations ± SEM (ms) *(% ± SEM)*	
			τ_1_	τ_2_	τ_3_	τ_4_	τ_1_	τ_2_
**HSP (α4β2)**_**2**_**β2-nAChR (HS isoform; concatenated subunits), L*ow [ACh] (1*.*3 μM)***								
(α4β2)_2_β2	5	1.0 ± 0.2	0.47 ± 0.09 *(14 ± 6%)*	23 ± 4 *(28 ± 7%)*	80 ± 30 *(58 ± 6%)*	Absent	1.1 ± 0.2 *(65 ± 6%)*	3.6 ± 0.4 ^Ϯ^ *(35 ± 6%*^Ϯ^*)*
**LSP (α4β2)**_**2**_**α4-nAChR (LS isoform; concatenated subunits), *low [ACh] (0*.*7 μM)***								
(α4β2)_2_α4	6	1.8 ± 0.4	0.65 ± 0.06 *(40 ± 10%)*	5.3 ± 0.9 *(25 ± 6%)*	110 ± 30 *(28 ± 7%)*	2100 ± 900 *(14 ± 3%)*	0.9 ± 0.3 *(70 ± 10%)*	3.5 ± 0.8^Ϯ^ *(40 ± 20%)*
**LSP (α4β2)**_**2**_**α4-nAChR (LS isoform; concatenated subunits), *high [ACh] (30 μM)***								
(α4β2)_2_α4	8	1.8 ± 0.3	0.8 ± 0.1 *(21 ± 4%)*	40 ± 10 *(33 ± 4%)*	100 ± 10 *(26 ± 2%)*	1500 ± 200 (34 ± 6%*)	0.35 ± 0.05* (71 ± 9%)	1.5 ± 0.3^Ϯ,^* (38 ± 9%^Ϯ^*)*

As for the same α4β2-nAChR isoforms expressed from individual subunits, patches containing HSP- or LSP α4β2-nAChR were exposed to macroscopic EC_50_ concentrations of ACh. For the HS isoform this was 1.3 μM. For the LS isoform, two different ACh concentrations (0.7 μM; low, or 30 μM; high) were used, to probe HS and LS phases of function. As in Table 1, all events were included in this initial analysis whether or not they were associated with bursts of openings. Data represent τ means ± SEM of parameters derived from individual patches during three or more individual experiments, with the number of patches noted in each case. All comparisons were performed using Student’s two-tailed, unpaired, t-tests (df = 8–14, significance noted when P < 0.05).

Closed duration distributions of patches containing HSP (α4β2)_2_β2-nAChR are best described using three components. Time constants associated with each component are presented, with percentages of events assigned to each component shown below each time constant; this convention is used throughout the table. As for the same isoform expressed using unlinked subunits (see Table 1), individual openings of HSP α4β2-nAChR exhibit a single small amplitude, but exhibit two distinct open durations, with τ_2_ having a significantly longer open duration (^Ϯ^), and a significantly larger percentage of the total HS isoform events occur during τ_1_ compared to τ_2_ (^Ϯ^).

For LSP (α4β2)2α4-nAChR, closed duration distributions are best described with four components, at both ACh concentrations applied. No statistically-significant changes in LS isoform closed dwell time parameters are observed between the two ACh concentrations, however a trend is evident for the closed time to shorten at the high ACh concentration. However, a significant increase in the percentage of LS-isoform events associated with the τ_4_ closed dwell-duration is revealed at the high ACh concentration, when compared to that at the low ACh concentration (*). Individual open times of this isoform are best fit with two significantly-distinct components at either ACh concentration (^Ϯ^). A larger proportion of total LS isoform events occur during τ_1_ compared to τ_2_ at the high ACh concentration (^Ϯ^). Increasing the ACh concentration significantly shortens the two open time constants associated with these two components (τ_1_ and τ_2_). No changes are noted in the proportions of short- vs. long-duration events as the ACh concentration is increased, however.

Concatenated α4β2-nAChR mean open duration data were best fit with two components, regardless of isoform or ACh concentration (Fig 5D–5F; Table 2). This number of open duration components is identical to that measured for the same isoforms expressed from unlinked subunits (Fig 4D–4F; Table 1). Also resembling the corresponding data from unlinked subunit HS (α4β2)_2_β2- and LS (α4β2)_2_α4-nAChR, when stimulated at the lower ACh concentration, HSP and LSP α4β2-nAChR showed very similar individual-event mean open times and distributions between τ_1_ and τ_2_ (Fig 5D and 5E; Table 2). In another striking point of similarity, exposure of LSP α4β2-nAChR to the higher ACh concentration resulted in a significant reduction in individual-event open times for both τ_1_ and τ_2_ (Fig 5E and 5F; Table 2).

### Burst analysis: Engagement of the third ACh binding site unique to LS (α4β2)_2_α4-nAChR at the high ACh concentration reduces time spent in closed conformations between bursts

We next studied additional burst-related properties. Biased ratios of unlinked subunits were chosen since they allowed more-rapid expression of HS (α4β2)_2_β2- or LS (α4β2)_2_α4- nAChR with corresponding benefits to oocyte health, and thus patch quality, in some batches of oocytes. We first analyzed closed times between bursts.

**Fig 6:**
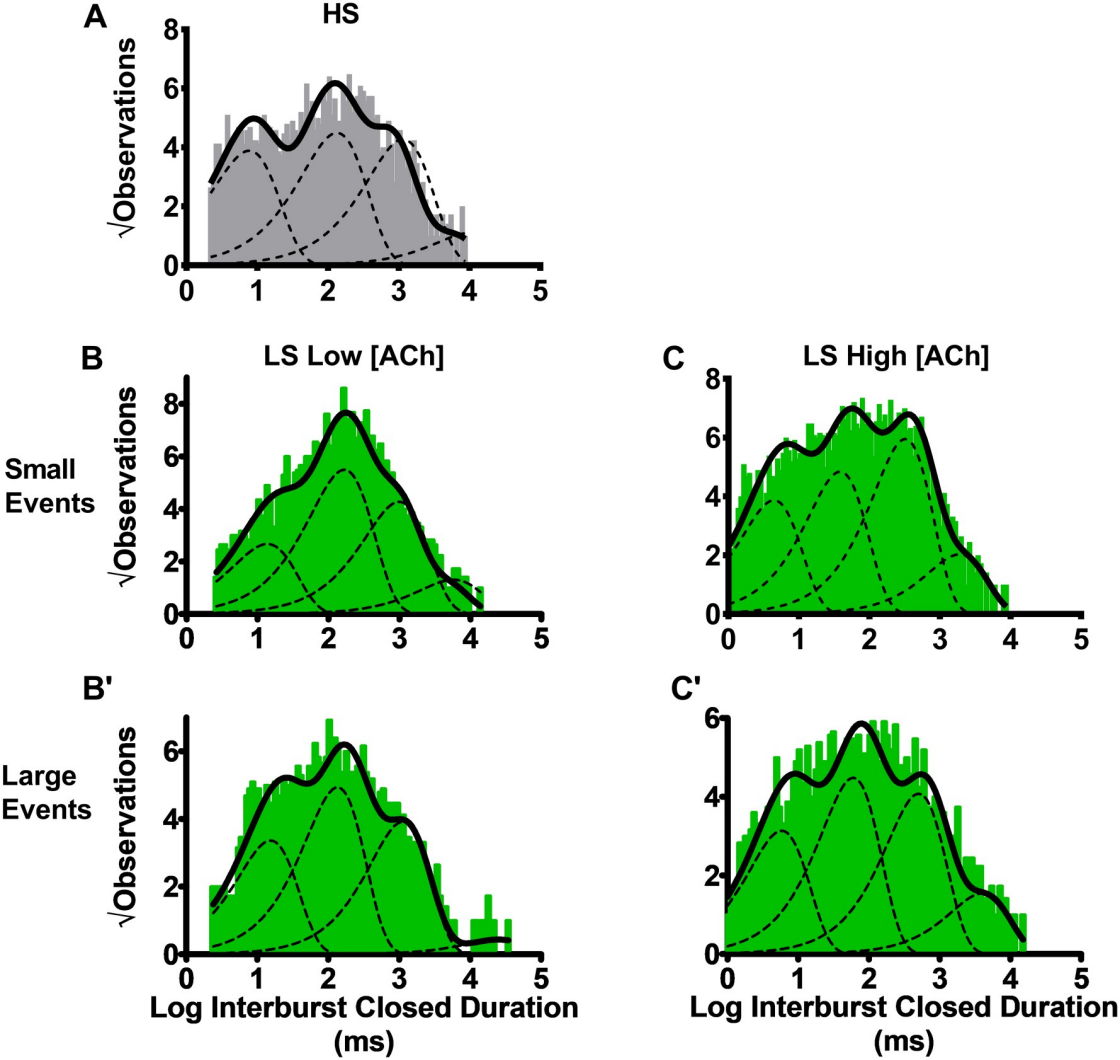
Closed durations between burst activity of human HS or LS α4β2-nAChR isoforms. Closed dwell durations between bursts of activity (interburst durations) were measured for HS or LS α4β2-nAChR isoforms expressed in *X*. *laevis* oocytes using unlinked subunits. **(A)** For HS (α4β2)_2_β2-nAChR, interburst closed durations evoked by 1.3 μM ACh were best described using four time constants. The same was true for closed durations between bursts of either small **(B)** or large amplitude **(B’)** events evoked from LS (α4β2)_2_α4-nAChR at a low ACh concentration (0.7 μM). Increasing the ACh concentration to 30 μM did not change the number of time constants associated with LS (α4β2)_2_α4-nAChR closed state intervals between bursts of small **(C)** or large **(C’)** events, but did result in significant shortening of the time constants in each case. Closed and open dwell duration pooled histograms are shown, which result from collection of data from 6–7 individual patches, across at least three separate experiments. The calculated τ values are summarized in Table 3, together with the statistical analyses applied.

For HS (α4β2)_2_β2-nAChR, the closed duration interburst histogram was best fit with four exponential components, with the longest lived state, τ_4_, having the smallest percentage of events (Fig 6A; Table 3). Analysis of LS (α4β2)_2_α4-nAChR was more complicated, since both small and large amplitude events were present. Significantly, bursts of events almost always were composed of either small or large events (i.e., >0.5% of bursts showed evidence of a transition between a small and large conductance state). This suggests that the two conductance states are associated with different functional states of LS (α4β2)_2_α4-nAChR. Accordingly, we separated small and large conductance bursts (see methods), and performed interburst analysis for each amplitude event class.

**Table 3:** Between-burst (interburst) closed durations, and within-burst (intraburst) open duration parameters for HS and LS α4β2-nAChR expressed using unlinked subunits.

Isoform	Interburst Closed Durations ± SEM (ms) *(% ± SEM)*								Individual Open Durations within bursts ± SEM (ms) *(% ± SEM)*		
	Small				Large				Small		Large
	τ_S1_	τ_S2_	τ_S3_	τ_S4_	τ_L1_	τ_L2_	τ_L3_	τ_L4_	τ_S1_	τ_S2_	τ_L1_
**HS (α4β2)**_**2**_**β2-nAChR (unlinked subunits), *low [ACh] (1*.*3 μM)***											
(α4β2)_2_β2	6.0 ± 0.1 *(24 ± 1%)*	85.45 ± 0.09 *(30 ± 2%)*	699.9 ± 0.1 *(36 ± 2%)*	6080 ± 1 *(9 ± 2%)*	Absent	Absent	Absent	Absent	0.65 ± 0.09 *(61 ± 6%)*	2.7 ± 0.1^Ϯ^ *(39 ± 6%*^Ϯ^*)*	Absent
**LS (α4β2)**_**2**_**α4-nAChR (unlinked subunits), *low [ACh] (0*.*7 μM)***											
(α4β2)_2_α4	12.4 ± 0.1 *(19 ± 1%)*	129.73 ± 0.09 *(40 ± 3%)*	692.4 ± 0.6 *(31 ± 3%)*	3618.2 ± 0.3 *(9 ± 3%)*	12.39 ± 0.08 *(27 ± 1%)*	135.26 ± 0.07**°** *(39 ± 2%)*	1109.3 ± 0.1**°** *(31 ± 2%)*	25128 ± 1**°** *(3 ± 2%)*	1.01 ± 0.05 *(52 ± 7%)*	Absent	1.66 ± 0.06 **°** *(48 ± 7%)*
**LS (α4β2)**_**2**_**α4-nAChR (unlinked subunits), *high [ACh] (30 μM)***											
(α4β2)_2_α4	4.42 ± 0.07* (23 ± 1%)	38.36 ± 0.07* (29 ± 1%)	321.4 ± 0.1* (36 ± 2%)	1891.5 ± 0.2* (12 ± 2%)	5.82 ± 0.08°,* (24 ± 1%)	59.88 ± 0.08°* (34 ± 2%)	504.2 ± 0.1°* (31 ± 2%)	4021.6 ± 0.2°* (12 ± 2%)	0.56 ± 0.05* (66 ± 8%)	Absent	0.86 ± 0.06°* (34 ± 8*%)

HS α4β2-nAChR function was stimulated at a low ACh concentration, corresponding to the macroscopic (α4β2)_2_β2-nAChR EC_50_ value at this isoform, whereas LS α4β2-nAChR function was stimulated at two different ACh concentrations (low and high), corresponding to the macroscopic EC_50_ values for HS and LS phases of function exhibited by this isoform, respectively. Data represent τ means ± SEM of parameters derived from individual patches during three or more individual experiments, with seven patches used in each case. All comparisons were performed using Student’s two-tailed, unpaired, t-tests (df = 12, significance noted when P < 0.05).

Closed dwell durations between bursts of HS isoform openings are best fit with four components. Individual openings within these bursts exhibit a single small amplitude, but exhibit two distinct open intraburst durations (^Ϯ^). This situation closely resembles that seen when analyzing all HS isoform open events, whether within bursts or not (see Table 1). Also similar to the earlier, less-selective analysis, a higher percentage of events are assigned to the shorter duration (τ_1_) population (^Ϯ^).

Bursts produced by LS α4β2-nAChR are either composed of small or large amplitude openings; parameters were calculated separately for the two populations. In both cases, closed dwell durations between bursts are best described with four components. At the low ACh concentration, large amplitude bursts exhibit longer closed dwell times between bursts than small amplitude bursts for the three longest interburst closed dwell times (τ_2_, τ_3_, and τ_4_ °). These differences were extended at the high ACh concentration, where large amplitude bursts retain longer interburst closed dwell durations compared to those seen between bursts of small amplitude events for all components (°). When comparing results for the two ACh concentrations used, the durations of all between-burst closed time components are significantly shortened at the high ACh concentration compared to the low ACh concentration. This is true both for closed dwell durations between bursts composed of small amplitude events, and for those between bursts of large amplitude events (*).

When considering individual openings within bursts of LS-isoform α4β2-nAChR, small amplitude openings exhibit a single mean duration. Large amplitude openings within bursts also are associated with a single mean duration, which is significantly longer than that of small amplitude openings. This difference is maintained between the two ACh concentrations applied (low ACh concentration small *vs*. large amplitude open duration within bursts and high ACh concentration small vs. large amplitude open duration within bursts: °). In addition, increasing the ACh concentration significantly reduces the duration of individual openings within both classes of bursts (small amplitude openings: τ_S1_ and large amplitude openings: τ_L1;_ *).

When LS (α4β2)_2_α4-nAChR were stimulated with a low ACh concentration (corresponding to the EC_50_ for HS phase macroscopic function), interburst closed duration histograms of small events were best described with four exponential components (Fig 6B; Table 3). This was the same as for the HS (α4β2)_2_β2-nAChR isoform at a corresponding ACh concentration (Fig 6A; Table 3). At this low agonist concentration, LS (α4β2)_2_α4-nAChR large event interburst closed durations were also best described with four components (Fig 6C; Table 3). Importantly, when LS (α4β2)_2_α4-nAChR were exposed to a low ACh concentration, interburst closed dwell times differed significantly between the small and large event bursts measured in the same patches. In particular, τ_3_ and τ_4_ were considerably longer between bursts of large events than between burst of small events (Table 3). This provides further evidence that small and large openings are associated with distinct functional states of LS (α4β2)_2_α4-nAChR.

When LS (α4β2)_2_α4-nAChR were stimulated with a high ACh concentration (corresponding to the EC_50_ for LS-phase macroscopic function), interburst closed durations were still best fit with four time components. However, all interburst τ values were significantly shortened at the higher ACh concentration. This was true both for bursts containing large openings, and those composed of small openings (Fig 6C and 6C’; Table 3). At this higher agonist concentration, large event bursts were again associated with significantly longer interburst closed durations than were small event bursts.

### Open events within small amplitude bursts of LS isoform (α4β2)_2_α4-nAChR are shorter in duration from those within large amplitude bursts, and both are shortened by increased ACh concentrations

The initial analysis of single-event open times did not distinguish between small and large amplitude openings of LS (α4β2)_2_α4-nAChR isoform. Studying only those events within bursts which, for LS (α4β2)_2_α4-nAChR, segregate into bursts containing events of only one amplitude, allowed us to make this distinction between small and large event open times with confidence.

**Fig 7:**
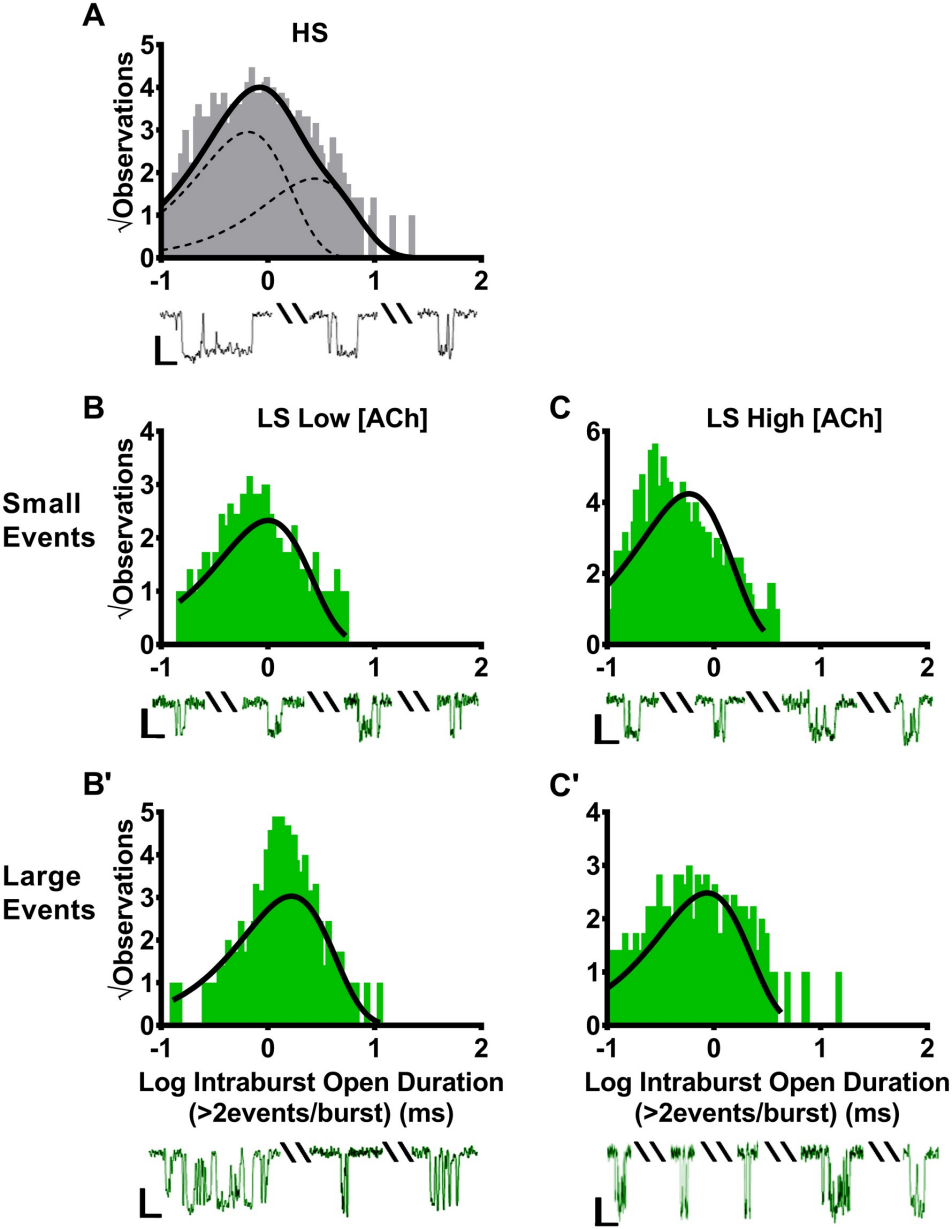
Durations of individual openings within bursts of small and large amplitude openings of human HS or LS α4β2-nAChR isoforms. HS or LS isoforms were expressed in *X*. *laevis* oocytes using unlinked subunits, and durations of individual openings within bursts (intraburst) of activity were measured. **(A)** HS (α4β2)_2_β2-nAChR stimulated with 1.3 μM ACh exhibited intraburst openings with a single, small, characteristic amplitude, as described previously. The open dwell durations associated with these openings were best described using a pair of time constants. For LS (α4β2)_2_α4-nAChR stimulated with a low ACh concentration (0.7 μM), bursts of either small **(B)** or large amplitude **(B’)** events were seen. Further, each type of burst was associated with a single, characteristic, intraburst duration of individual openings. The open durations for individual events within small amplitude bursts were significantly shorter than those associated with individual events within large amplitude bursts. When the ACh concentration was increased to 30 μM, the same general outcome was found. Intraburst open durations of individual openings of LS (α4β2)_2_α4-nAChR within small **(C)** and large amplitude **(C’)** bursts remained associated with single time constants, and the durations of small amplitude individual openings continued to be shorter than those of large amplitude individual openings. However, the durations of both event classes were significantly shortened in the presence of the higher ACh concentration. Histogram panels each show pooled data, which were collected from 6–7 individual patches, across at least three separate experiments. Representative traces of each category of bursts are shown below the corresponding histogram panels. Scale bars are 1 pA in height, and 10 ms in width. The calculated τ values are summarized in Table 3, together with the statistical analyses applied.

Mean open-time durations within bursts were initially determined for HS (α4β2)_2_β2-nAChR. Since openings for HS (α4β2)_2_β2-nAChR occur as only a single amplitude population, this provides a valuable control for internal consistency. As would be expected from the initial all-event analysis, open durations within bursts (intrabursts) fell into two populations (Fig 7A). For open events within bursts, these two populations were associated with very similar values of τ_1_ and τ_2_, and percentage distributions between the shorter and longer open time populations (Table 3), to those calculated across all events (compare to Table 1).

Reassured by this demonstration of internal consistency, we next analyzed durations of individual openings within bursts of either small- or large-events arising from LS (α4β2)_2_α4-nAChR. This analysis was first performed for bursts elicited by the low ACh concentration. Surprisingly, as shown in Fig 7B and B’, the durations of individual openings within LS bursts segregated with small or large amplitude events. Specifically, small amplitude intraburst open durations were associated with a single τ value, which was significantly shorter than the single τ value associated with large amplitude intraburst open durations (Table 3). This segregation was maintained when LS (α4β2)_2_α4-nAChR were stimulated with the higher ACh concentration (Fig 7C and 7C’). However, the mean open times of individual events within bursts of both small and large individual openings were significantly reduced (approximately halved) at the higher agonist concentration (Table 3). Notably, similar trends were seen in the earlier analysis of all individual openings arising from LS (α4β2)_2_α4-nAChR activation (Table 1), which did not distinguish between small and large amplitude openings. However, values from this segregated analysis are more definitive.

### Site-directed mutagenesis confirms importance of the α4(+)/(-)α4 agonist binding site for elevated ACh concentration effects on closed durations between bursts of LS isoform (α4β2)_2_α4-nAChR

The preceding findings suggested that agonist binding to the α4(+)/(-)α4 site found only in LS (α4β2)_2_α4-nAChR significantly reduces between-burst closed durations. To test this inference directly, we proceeded to disrupt *only* the α4(+)/(-)α4 site, which required use of a concatemeric nAChR construct. We have previously shown that introducing an alanine mutation at position W182 in only the α4 subunit at position five of the LSP concatemer (β2-α4-β2-α4-α4[W182A]; LSP-α4p5W182A) severely hampers function of the α4(+)/(-)α4 ACh binding site, significantly reducing the macroscopic LS-phase response of the mutated receptor [27]. Therefore, the LSP α4p5W182A-nAChR should have substantially reduced single channel property changes between low and high ACh concentrations, compared to those previously observed with unmodified LSP (α4β2)_2_α4-nAChR.

**Fig 8:**
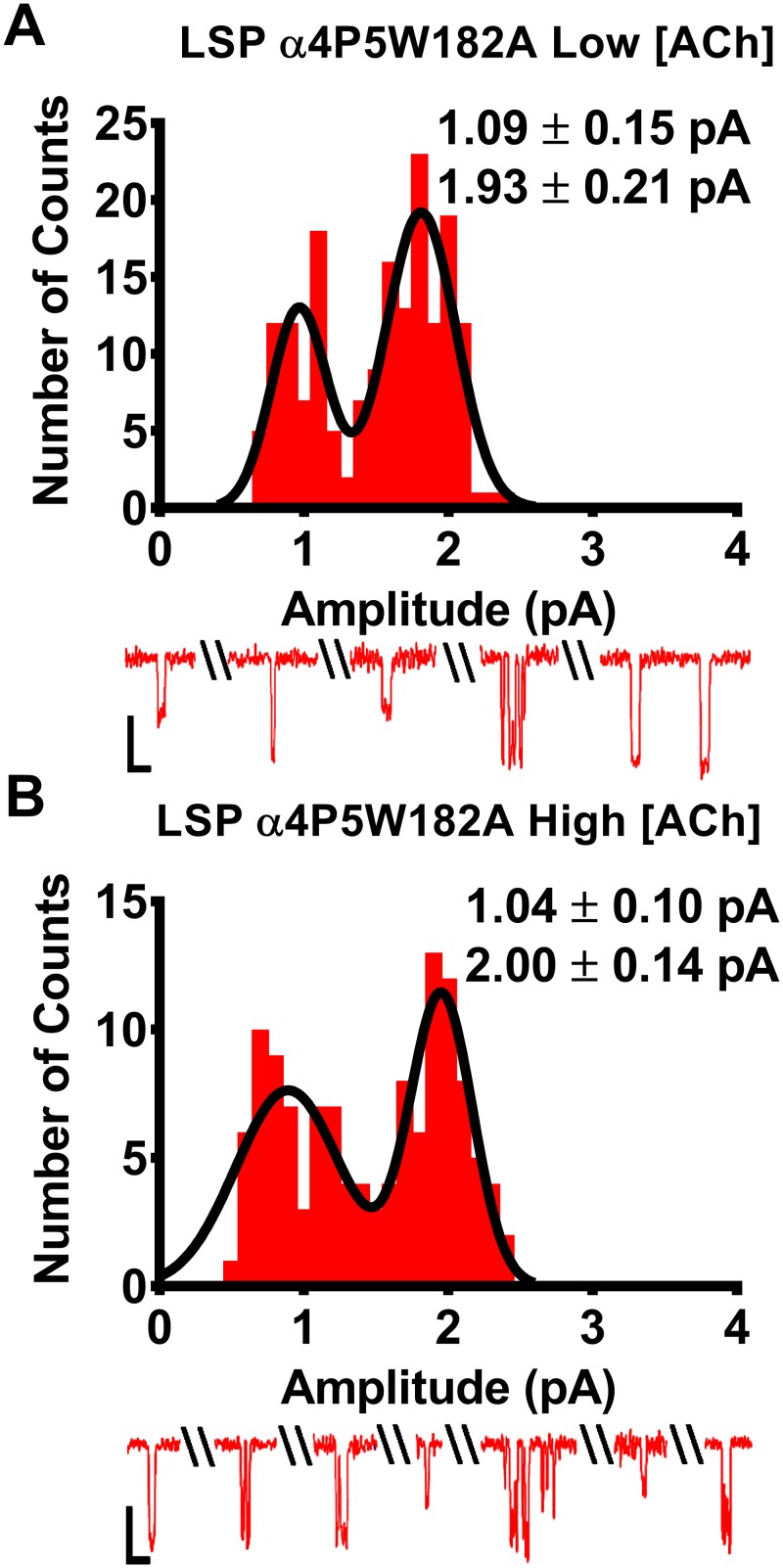
ACh unitary amplitudes and conductances associated with human LSP α4P5W182A-nAChR expressed in *X*. *laevis* oocytes from pentameric, fully-concatenated constructs. All statistical comparisons for this figure were performed using two-tailed unpaired Student’s t-tests, df = 11–12, significant differences were noted at P < 0.05. **(A)** LSP α4P5W182A-nAChR were stimulated with a low ACh concentration (0.7 μM ACh). Evoked unitary responses produced openings that fell into two amplitude classes (small and large). These were statistically indistinguishable from those observed from LSP α4β2-nAChR constructs that did not harbor the α4P5W182A, when these were stimulated with the same concentration of ACh. (Fig 3B). **(B)** LSP α4P5W182A-nAChR were stimulated with a high (30 μM) ACh concentration. Single-channel openings displayed two amplitude classes that were indistinguishable from those in panel A using a low ACh concentration, and those of LSP-nAChR produced using the same high ACh concentration (Fig 3C). Example traces are shown below panels (A) and (B), demonstrating a mixture of individual openings at each amplitude level, and short bursts of activity followed by longer periods of inactivity. Values are given as mean ± S.E.M. Amplitude Data were collected across a minimum of three separate experiments from 7 patches, across a minimum of three separate experiments. All recordings were performed 3—6d following cRNA injection.

Single channel openings of LSP α4p5W182A-nAChR exhibited two unitary amplitudes at either low (Fig 8A) or high (Fig 8B) ACh concentrations. The observed values, and their consistency between the two ACh concentrations used, closely match those for unmodified LS (α4β2)_2_α4-nAChR expressed using either unlinked or concatenated subunits (Figs 2B and 2C or 3B and 3C, respectively). Two different conductances were measured for the LSP α4p5W182A-nAChR, which also closely matched those determined at non-mutant LSP α4β2-nAChR (Fig 3E). Accordingly, we analyzed bursts containing small or large openings separately, as described for unmodified LS-(α4β2)_2_α4-nAChR in the preceding section.

**Fig 9:**
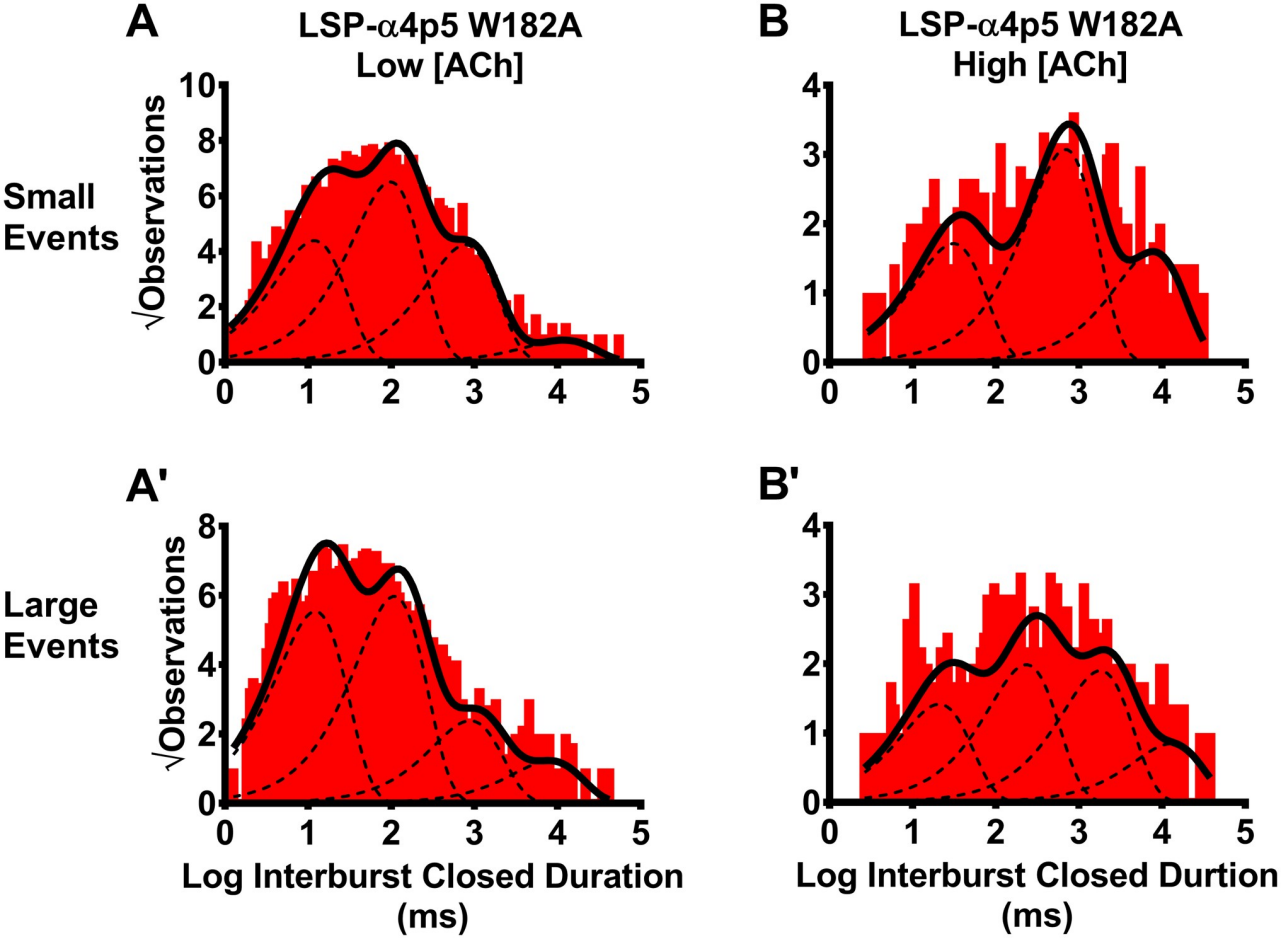
Closed dwell durations between small and large amplitude bursts of human LSP-α4p5W182A-nAChR. LSP (α4β2)_2_α4-nAChR were expressed in *X*. *laevis* oocytes from a concatenated cDNA construct in which the critical tryptophan-182 amino-acid was mutated to alanine (W182A) in only the low-affinity α4/α4-interface agonist binding site (red bars). Closed durations between bursts of activity in the presence of a low ACh concentration (0.7 μM) were determined for both small **(A)** and large amplitude **(A’)** bursts. As for the parent LSP construct, interburst closed durations at the low ACh concentration were best described using four time constants, regardless of whether small or large amplitude bursts were considered. The effects of changing to a higher ACh concentration (30 μM) on interburst closed durations differed between the two amplitude classes of bursts. For bursts of small amplitude events **(B)**, the number of time components was reduced to three, likely due to disappearance of the second-shortest time component (τ_S2_). In this interpretation, the shortest time constant increased significantly while small, but significant, shortenings of the two longest time constants (τ_S3_, τ_S4_) were also seen. These shortenings were much less dramatic than those observed for the parent LSP construct (compare to Fig 6). For bursts of large amplitude events **(B’)**, the increased ACh concentration did not alter the number of interburst closed dwell duration components detected. However, each time constant was significantly lengthened; this is the opposite of the behavior of the parent LSP construct. Data were collected from 7 individual patches, across at least three separate experiments, for each panel of the Fig. The calculated τ values are summarized in Table 4, together with the statistical analyses applied.

We looked first at effects of the LSP α4p5W182A mutation on closed durations between bursts of small amplitude events. When stimulated at a low ACh concentration, these were best fit with four closed components (Fig 9A; Table 4). This is the same number of components as found for unmodified LS (α4β2)_2_α4-nAChR (Table 3). As predicted, moving to the higher ACh concentration no longer produced a concerted shortening of intervals between bursts containing small events. Instead, a more-complex picture was revealed. At the higher ACh concentration, the LSP α4p5W182A-nAChR small event closed dwell time distribution was best fit with three components, likely due to loss of the τ_2_ component (Fig 9B). In this interpretation, the shortest closed-time component (associated with τ_1_) instead was *lengthened* significantly. By contrast, the remaining components shortened significantly (Table 4), but to a much smaller proportion than was observed for unmodified LS (α4β2)_2_α4-nAChR (Table 3). We note that an alternative interpretation is possible: that all of the values of τ_1_, τ_2_, and τ_3_ are increased, with the absence of a fourth component resulting from there being too few events detectable within the recording time available before significant functional run-down of the patches occurs.

**Table 4:** Between-burst (interburst) closed durations, and within-burst (intraburst) open duration parameters associated with burst activity of LSP-α4p5W182A-nAChR.

Isoform	Number of Patches	Closed Durations ± SEM (ms) *(% ± SEM)*								Individual Open Durations within bursts ± SEM (ms) *(% ± SEM)*		
		Small				Large				Small		Large
		τ_S1_	τ_S2_	τ_S3_	τ_S4_	τ_L1_	τ_L2_	τ_L3_	τ_L4_	τ_S1_	τ_S2_	τ_L1_
**LSP-α4p5W182A-nAChR (concatenated subunits), *low [ACh] (0*.*7 μM)***												
(α4β2)_2_α4 p5W182A	7	11.88 ± 0.07 *(28 ± 1%)*	97.46 ± 0.06 *(41 ± 1%)*	783.3 ± 0.1 *(27 ± 1%)*	11801.0 ± 0.3 *(5 ± 1%)*	12.19 ± 0.06° *(37 ± 2%*°*)*	108.51 ± 0.08° *(40 ± 2%)*	885.6 ± 0.2° *(16 ± 2%*°*)*	8138.9 ± 0.3° *(8 ± 2%)*	0.76 ± 0.06 *(57 ± 2%)*	Absent	2.12 ± 0.05° (30 ± 10*%)
**LSP-α4p5W182A-nAChR (concatenated subunits), *high [ACh] (30 μM)***												
(α4β2)_2_α4 p5W182A	7	30.8 ± 0.1* (27 ± 2%)	Absent	687.3 ± 0.1* (48 ± 3%)	7814.5 ± 0.1* (25 ± 3%)	20.8 ± 0.2°^,^* (23 ± 3%)	231.5 ± 0.2* (32 ± 4%)	1805.7 ± 0.2°^,^* (31 ± 4)	12821.8 ± 0.4°^,^* (14 ± 4%)	1.07 ± 0.08* (63 ± 6*%)	Absent	2.33 ± 0.06°,* (37 ± 6%)

An α4W182A mutation was placed within the α4(+)/(-)α4 agonist binding site of LSP α4β2-nAChR, which significantly reduces the ability of this site to evoke LS phase macroscopic function. LSP α4p5W182A-nAChR patches were stimulated at two different ACh concentrations (low and high), corresponding to the macroscopic EC_50_ values for HS and LS function. As for non-mutated LS α4β2-nAChR, bursts arising from LSP α4β2-nAChR are either small or large amplitude, and parameters were calculated separately for the two populations. Data represent means ± SEM of parameters derived from 7 individual patches, as noted in the table, recorded during three or more individual experiments. All comparisons were performed using Student’s two-tailed, unpaired, t-tests (df = 12, significance noted when P < 0.05).

At the low ACh concentration, closed dwell durations between bursts of small amplitude events are best fit with four components, as are those between bursts of large amplitude events. Similar to the situation with the LSP parent construct, large amplitude bursts produced by LSP-α4p5W182A-nAChR generally exhibit longer closed dwell times between bursts than were observed between small amplitude bursts (τ_1_, τ_2_, and τ_3_; °). The one exception noted is that the longest closed dwell time between small amplitude bursts at the low ACh concentration is significantly longer that measured between large amplitude bursts (τ_4_;°). When moving to the high ACh concentration, only three components are needed to fit the distribution of closed dwell durations between bursts of small amplitude events, due to the loss of τ_S2_. In contrast, closed dwell durations between bursts of large amplitude events remain best-fit by four components. When comparing the three remaining mean closed interburst dwell duration components observed between small amplitude bursts at the high ACh concentration to their large amplitude counterparts: The shortest component τ_1_ is significantly longer between bursts of small vs. large amplitude events (°). For the remaining two interburst closed dwell duration components, the opposite outcome is observed (longer between bursts of large amplitude events, similar to observations at the un-mutated LSP construct; τ_3_ and τ_4_;°). Significant differences are observed when comparing interburst closed duration components between the two ACh concentrations used. In the case of interburst closed dwell durations of small amplitude openings: τ_S1_ is significantly lengthened at the higher ACh concentration (*), whereas τ_S3_ and τ_S4_ are both significantly shortened (*). As noted earlier, the τ_2_ component associated with small amplitude closed interburst durations stimulated by the low ACh concentration is no longer observed at the high ACh concentration. In the case of closed-dwell durations between bursts of large amplitude openings, all four components are significantly lengthened in the presence of high vs. low ACh (τ_L1_, τ_L2_, τ_L3_, and τ_L4_;*).

Regarding individual openings within bursts of LSP α4p5W182A-nAChR, small amplitude openings exhibit a single mean duration. Large amplitude openings within bursts also are associated with a single mean duration, which is significantly longer than that for small amplitude openings. This difference is maintained between the two ACh concentrations applied (low ACh concentration small *vs*. large amplitude open duration within bursts and high ACh concentration small vs large amplitude open duration within bursts; °). In addition, increasing the ACh concentration significantly increases the duration of individual openings within both classes of bursts (small- and large-amplitude openings: τ_1_ and τ_2_; * P < 0.05, df = 12).

Closed durations between bursts of LSP α4p5W182A-nAChR large amplitude events were analyzed next. When LSP α4p5W182A-nAChR were stimulated at the low ACh concentration, the large amplitude interburst closed duration histograms were best fit with four components (Fig 9A’; Table 4). This again matched the findings for unmodified LS (α4β2)_2_α4-nAChR (Table 3). Also as predicted, stimulation with the higher ACh concentration no longer resulted in a systematic reduction in closed times between large event bursts, as observed for unmodified LS (α4β2)_2_α4-nAChR. Instead, all four τ values showed significant *increases* in duration (Fig 9B’, Table 4).

### Site-directed mutagenesis confirms importance of the α4(+)/(-)α4 agonist binding site for elevated ACh concentration effects on mean open time durations of individual openings within between bursts of LS isoform (α4β2)_2_α4-nAChR

**Fig 10:**
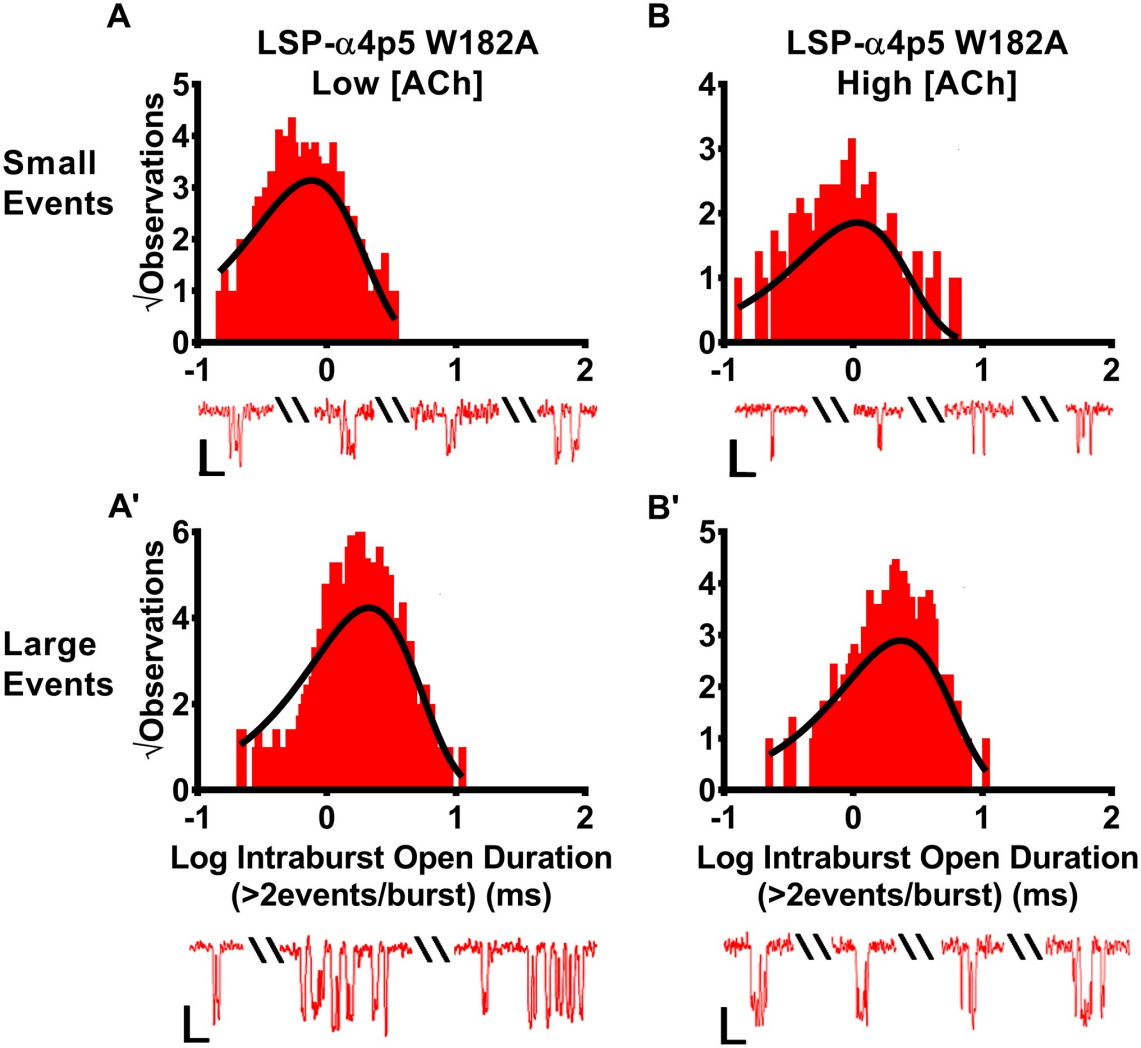
Durations of individual openings within small or large amplitude event bursts of human LSP-α4p5W182A-nAChR. LSP (α4β2)_2_α4-nAChR harboring the α4p5W182A mutation, which significantly reduces macroscopic LSP function, were expressed in *X*. *laevis* oocytes using a concatenated cDNA construct. Similar to the parent LSP construct, when stimulated with a low ACh concentration (0.7 μM), bursts of either small **(A)** or large amplitude **(A’)** events were seen, and individuals openings within each type of burst were associated with a single, characteristic, open duration. Also as seen for the parent construct, the intraburst open durations for individual events within small amplitude bursts were significantly shorter than those associated with individual events within large amplitude bursts. In a further similarity to the parent construct, when the ACh concentration was increased to 30 μM, intraburst open durations of individual openings of LSP (α4β2)_2_α4-nAChR within small **(B)** and large amplitude **(B’)** bursts remained associated with single time constants, and the individual openings with small amplitude bursts continued to be shorter than those within large amplitude bursts. However, in direct opposition to the outcome for the unmutated LSP construct, durations of both small and large amplitude individual openings were significantly lengthened in the presence of the higher ACh concentration. Intraburst open duration τ values have been inserted into each panel to facilitate interpretation. Representative examples of bursts obtained from this mutant construct are shown below the corresponding histogram panels. Scale bars are 1 pA in height, and 10 ms in width. Data were collected from 7 individual patches, across at least three separate experiments. The calculated τ values are summarized in Table 4, together with the statistical analyses applied.

In addition to significantly reducing closed times between bursts of small and large openings of unmodified LS (α4β2)_2_α4-nAChR, our earlier findings suggested that ACh binding to the lower-affinity α4/α4 site reduced the duration of individual open events within bursts. The LSP α4p5W182A construct was also used to address this inference, with the prediction that damaging the α4(+)/(-)α4 agonist binding pocket would substantially block this effect. Similar to unmodified LS (α4β2)_2_α4-nAChR, ACh-stimulated, within-burst, small and large amplitude open events recorded from LSP α4p5W182A-nAChR were each best fit with a single time constant. This was true whether openings were evoked using either a low or high ACh concentration (Fig 10, Table 4). In a further point of similarity with unmodified LS (α4β2)_2_α4-nAChR, the mean durations of individual openings within small amplitude bursts of LSP α4p5W182A-nAChR were shorter than those within large amplitude bursts, when both were measured at the low ACh concentration. However, in strong contrast to the effect on the unmodified LS (α4β2)_2_α4-nAChR, increasing the ACh concentration did not shorten open times for individual events within bursts arising from LSP α4p5W182A-nAChR. Instead a slight, but significant, increase in intraburst open durations was observed (Table 4). This finding again matches the predicted outcome.

### α4(+)/(-)α4 agonist binding site engagement at elevated ACh concentration increases bursting of both low- and high-conductance openings of LS (α4β2)_2_α4-nAChR.

We next examined further burst-related properties of LS (α4β2)_2_α4-nAChR expressed from unlinked subunits. Single-channel responses were again compared between low and high concentrations of ACh.

**Fig 11:**
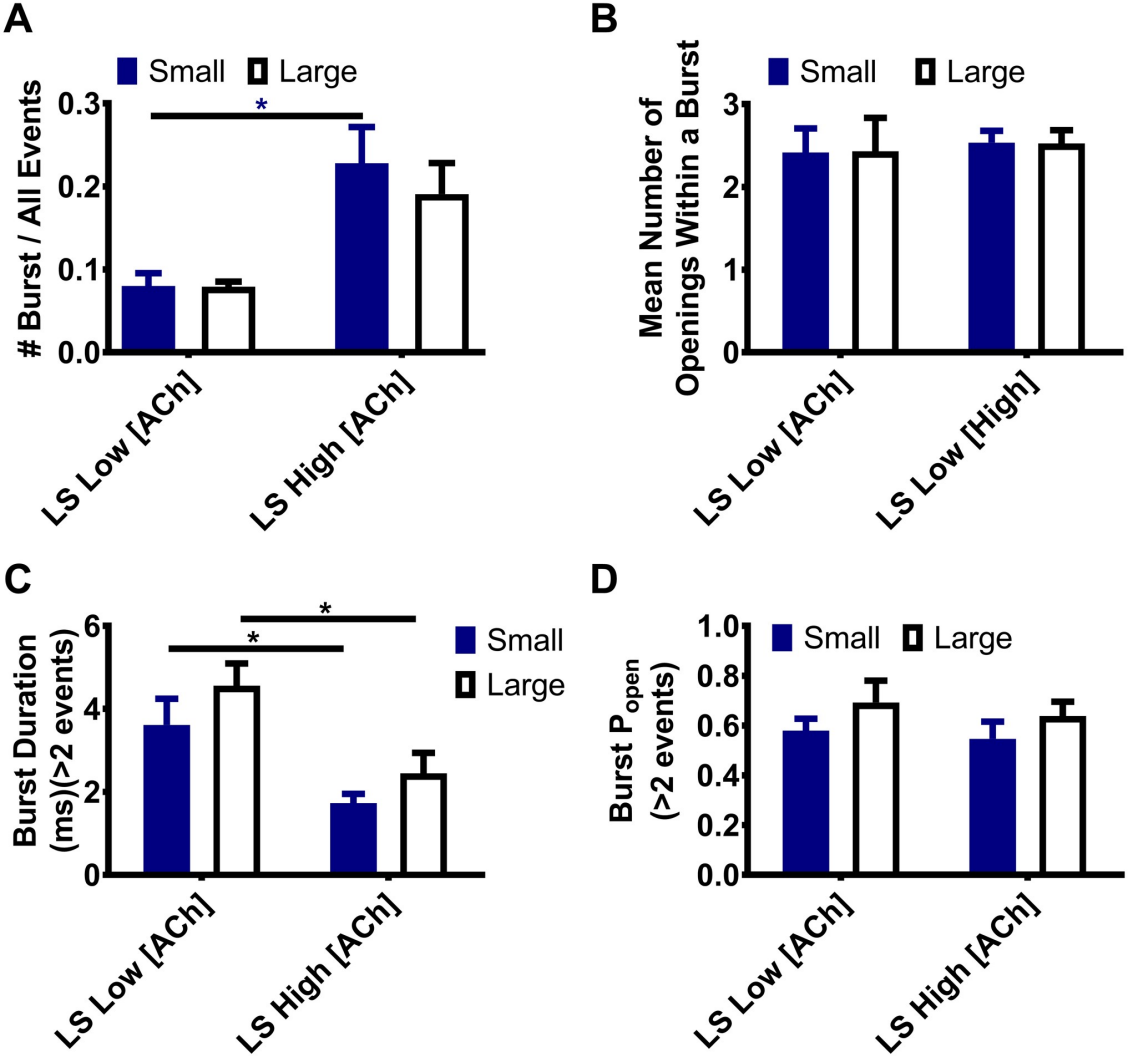
Human LS α4β2-nAChR small and large amplitude burst parameters. LS α4β2-nAChR isoforms were expressed in *X*. *laevis* oocytes using unlinked subunits and burst parameters were measured. Comparisons were made using Student’s two-tailed, unpaired, t-tests (df = 9–11, significant differences noted at p < 0.05). **A)** The mean proportion of small or large amplitude openings falling within bursts, compared to the total number of each type of open event, was determined at a low ACh (0.7 μM) and a high ACh (30 μM) concentration. For both small and large amplitude openings, the proportion of events within bursts of the corresponding amplitude appeared to increase with application of a high ACh concentration. A significant increase in the small amplitude burst proportion was observed with a high ACh concentration compared to the low ACh concentration (*). **B)** The mean number of openings per burst of LS isoform small or large amplitude openings was determined at both the low and high ACh concentrations. Altering the concentration of ACh had no significant effect on the numbers of openings within either class of bursts. **C)** The durations of LS isoform small or large amplitude bursts were measured at the low and high ACh concentrations. Increasing the ACh concentration significantly shortened the duration of both small and large amplitude bursts (*). **D)** The P_open_ within bursts was indistinguishable between bursts of small or large amplitude events, and was not significantly altered by an increase in applied ACh concentration from 0.7 μM to 30 μM.

The proportion of events that fell inside a burst significantly increased at the higher ACh concentration for small amplitude events, with a trend for increased large amplitude events (low ACh concentration small amplitude bursts 0.080 ± 0.015, large amplitude bursts 0.079 ± 0.006; high ACh concentration small amplitude bursts 0.228 ± 0.044, large amplitude bursts 0.191 ± 0.037; Fig 11A). This increase was predicted by earlier findings showing that the proportion of within-burst closed events (associated with τ_1_ closed duration in Table 1) increased at the higher ACh concentration. Surprisingly, the high concentration of ACh did not significantly increase the proportion of large amplitude openings uniquely associated with the LS isoform compared to the small amplitude events (51 ± 7% and 55 ± 5% of events were of small amplitude at 0.7 μM ACh and 30 μM ACh, respectively). Interestingly, the number of open events per burst was unchanged at the higher ACh concentration (Fig 11B). This was true for bursts of either small or large amplitude events (low ACh concentration small amplitude bursts 2.4 ± 0.3, large amplitude bursts 2.4 ± 0.4; high ACh concentration small amplitude bursts 2.5 ± 0.1, large amplitude bursts 2.5 ± 0.2). As would be predicted from the fact that numbers of events within bursts remained the same at low or high ACh concentrations, but the durations of openings and closings were shorter at the higher ACh concentration, burst durations were significantly shortened in the presence of high ACh (low ACh concentration: small amplitude bursts 3.6 ± 0.6, large amplitude bursts 4.6 ± 0.5; high ACh concentration: small amplitude bursts 1.7 ± 0.2, large amplitude bursts 2.4 ± 0.5; Fig 11C). The simultaneous shortening of both open and closed durations also resulted in unchanged P_open_ values within bursts of small or large amplitude events, between the two ACh concentrations (low ACh concentration small amplitude bursts 0.58 ± 0.05, large amplitude bursts 0.69 ± 0.09; high ACh concentration small amplitude bursts 0.55 ± 0.07, large amplitude bursts 0.64 ± 0.06; Fig 11D). Overall, the effects of increased ACh concentration on LS (α4β2)_2_α4-nAChR were to increase the proportions of openings found within bursts of either small or large amplitude openings (indicative of a shift to increased bursting behavior), while simultaneously shortening the durations of individual openings within bursts.

## Discussion

Previous single-channel studies of α4β2-nAChR function have usually examined populations with mixed stoichiometry. These have reported the existence of two, or occasionally three, distinct conductance states [23, 46–53]. These findings are broadly consistent with our own, which show a single conductance state associated with the HS α42-nAChR isoform, and two conductance states associated with the LS α4β2-nAChR isoform (one similar but not identical to that of the HS isoform, and one larger). The specific conductance values measured in this study also fall within the range of values reported previously, using nAChR subunits from a range of species, expression backgrounds, recording conditions (such as ionic strength and calcium concentrations), and patch configurations (see preceding citations). Importantly, the lower conductance associated with small amplitude LS α4β2-nAChR isoform (expressed using unlinked or linked subunits) openings was significantly different from the single conductance state measured for HS α4β2-nAChR (expressed using unlinked or linked subunits), proving that its appearance was not simply due to isoform contamination. Equally importantly, bursts containing multiple open amplitudes were extremely rare (< 0.05% of all events analyzed) and, in any case, were excluded from the analysis. As a result, the higher conductance state uniquely associated with LS (α4β2)_2_α4-nAChR cannot be an artifact produced by multiple, smaller-conductance, channel openings occurring simultaneously. Further evidence that small and large amplitude openings of the LS isoform correspond to distinct states of the receptor is provided by the fact that each is associated with a different, defined, open duration (with the large-conductance openings having longer open times for both ACh concentrations tested).

Importantly, two recent publications have also reported that a high conductance state arises uniquely from LS (α4β2)_2_α4-nAChR [38, 54]. In one case [38], biased injection ratios of unlinked subunits were used, and increased agonist concentrations were seen to increase the prevalence of high-conductance, high amplitude openings over low amplitude events. This led to a conclusion that the larger amplitude state arose from the LS isoform, in agreement with our own findings. However, only large amplitude events were used in their subsequent kinetic modeling, which our findings indicate may be an over-simplification. In the other case [54], the authors selected only “single channel currents that reached full amplitude” when analyzing the behavior of LS (α4β2)_2_α4-nAChR, eliminating smaller openings from further consideration when examining behavior of this isoform. Our own results differ in the critical respect that both low- and high-conductance events, with well-defined amplitudes, were noted to arise from pure populations of LS (α4β2)_2_α4-nAChR. This phenomenon was seen even when stimulated with a low ACh concentration, which should only minimally engage the lower affinity α4(+)/(-)α4 subunit interface agonist binding site uniquely found in the LS isoform [27–29]. Accordingly, access to the higher-conductance state of LS (α4β2)_2_α4-nAChR does not appear to require agonist binding at the α4(+)/(-)α4 subunit interface. This point is reinforced by the fact that LSP-α4p5W182A-nAChR also exhibit both low- and high-conductance states, despite harboring a mutation that damages the α4(+)/(-)α4 agonist binding site. Thus, the mere presence of an α4 subunit in “position 5” alters single channel properties. This is consistent with a fundamental effect of receptor structure, rather than emergence of the higher conductance state due to agonist occupancy of the α4(+)/(-)α4 subunit interface. Possible contributors to this phenomenon include 1) presence of alternate α4 subunit sequence elements directly lining the ion-conduction pathway, 2) substitution of an α4 subunit second intracellular loop for a shorter β2 subunit second cytoplasmic domain, or 3) an altered environment neighboring the conserved α4(+)/(-)β2 subunit interfaces which host the orthosteric agonist binding sites in both isoforms. The current study is not capable of distinguishing between these scenarios, although we note that our previous study indicated that the identity of subunits neighboring the otherwise-equivalent α4(+)/(-)β2 agonist sites modifies their contributions to nAChR activation (as measured at the macroscopic level), and that E-loop residues are an important contributor to this neighbor effect [31]. However, the recent publication of cryo-EM structures of both HS (α4β2)_2_β2-nAChR and LS (α4β2)_2_α4-nAChR isoforms do provide some valuable insights [55]. Addressing point 1), these structures show that the ion-permeation pathway of the LS (α4β2)_2_α4-nAChR isoform is more strongly electronegative throughout, which is compatible with a higher cation conductance. As noted by [55], the constructs used in their structural studies lack much of the second intracellular loop, so they are not able to address the preceding point 2) (the influence of this region, which can also change the overall conductance of related LGICs [56]). Importantly, the new cryo-EM structural data do show that the packing at subunit interfaces, tapering within the ion-permeation pathway, and relative orientations of subunits are significantly different between the two α4β2-nAChR isoforms [55]. This information indirectly addresses the preceding point 3, suggesting possibilities for the isoforms to access different open conformations that may in turn produce the alternate open conductance states that we have measured in this study.

Nevertheless, our initial assumption was that large amplitude openings of the LS (α4β2)_2_α4-nAChR isoform would at least be encouraged by increasing the ACh concentration sufficiently to engage the additional α4(+)/(-)α4 subunit interface site. However, the proportion of small- *vs*. large-amplitude openings was not changed by this pharmacological manipulation (Fig 11A). Instead, two major kinetic effects of increased ACh concentration on LS (α4β2)_2_α4-nAChR function were to 1) decrease single-channel closed dwell durations between bursts and 2) to shorten the duration of individual open events within bursts. We do note that the observation of shorter closed dwell durations between bursts needs to be interpreted with caution, since the exact number of receptors in each patch is not known. However, in this study, we compared patches containing nAChR expressed from the same constructs, in the same expression system, for the same length of time, and outliers with exceptionally low or high amounts of activity were eliminated from the analysis, all of which should reduce variability in number of receptors per patch. Furthermore, by use of the α4p5W182A mutation to specifically cripple the α4(+)/(-)α4 low-affinity agonist-binding site within the LSP concatemer [27], we were able to confirm the site’s effect on shortening both receptor closed dwell times between bursts and also the duration of individual open events within bursts. Specifically, the mutation consistently disrupted the kinetic differences associated with stimulating LS (α4β2)_2_α4-nAChR at HS *vs*. at LS ACh concentrations. These observations definitively link ACh engagement of the α4(+)/(-)α4-interface agonist binding site with the characteristic changes in single-channel kinetics associated with LS (α4β2)_2_α4-nAChR following exposure to high ACh concentrations. This outcome is consistent with allosteric potentiation of agonist actions at α4(+)/(-)β2 subunit interfaces, rather than an independent induction of channel opening due to additional occupancy of the α4(+)/(-)α4 subunit interface.

The other major effect observed on LS isoform function at the high ACh concentration was that bursts became more prevalent (as shown by the increased proportion of openings within bursts). Combined with the just-noted concerted shortening of closed durations between bursts, the overall effect of high ACh concentrations at LS (α4β2)_2_α4-nAChR can be summarized as enhancement of bursting, leading to enhanced function-per-receptor. These findings are compatible with the inference that agonist binding at the α4(+)/(-)α4 interface produces increased bursting due to preferred entry into an intermediate, pre-opening, “flip state” [38, 57].

**Fig 12:**
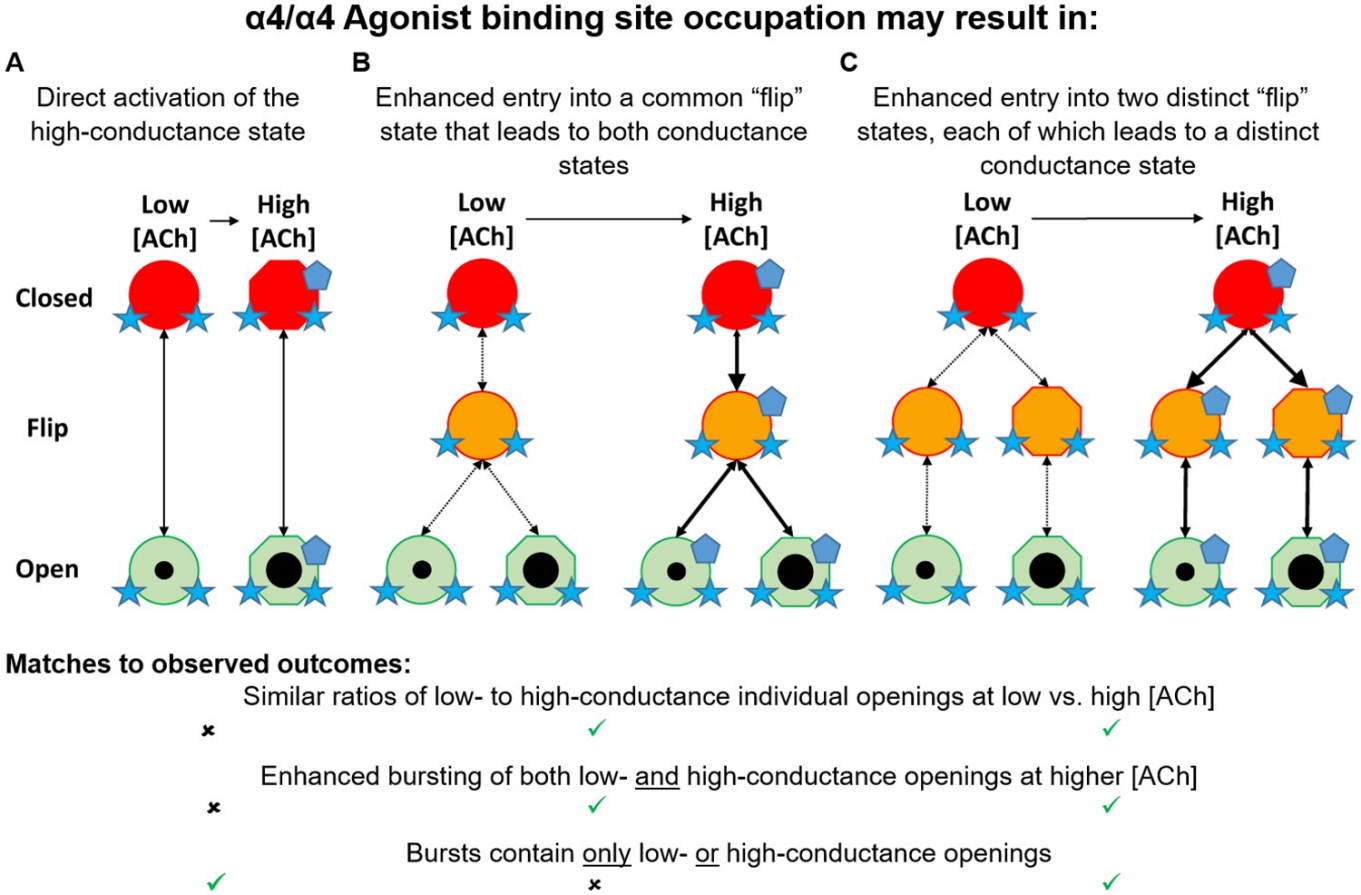
Comparison of mechanisms considered to explain the observed functional effects of ACh binding at the α4(+)/(-)α4 agonist binding site within LS-isoform α4β2-nAChR. In all panels, red shapes denote receptors in a closed state while bound by ACh. Intermediate “flip” states (also closed) are represented by orange shapes, while open states are indicated with green shapes (low- and high-conductance open states are shown as small and large black dots, respectively, within these green shapes). Where each model suggests emergence of a distinctly-different pathway, shapes change from circles to octagons. Receptor transitions between states are indicated with double-ended arrows, and bolder lines indicate increased probability of that transition occurring. ACh bound to the pair of canonical, high-affinity, α4(+)/(-)β2 agonist binding pockets is symbolized by blue stars, and a blue pentagon represents ACh bound to the low-affinity α4(+)/(-)α4 agonist binding site uniquely found within LS-isoform α4β2-nAChR. **A)** Since high-conductance openings are uniquely associated with LS-isoform α4β2-nAChR, the simplest potential explanation is that ACh binding to the α4(+)/(-)α4 agonist binding site directly produces entry into the high-conductance state. While this mechanism can explain the observation that almost all bursts contain either low or high-conductance openings, it is refuted since it predicts that the ratio of high- to low-conductance individual openings (and bursts thereof) should rise as the ACh concentration is increased. **B)** In contrast, a mechanism in which ACh binding to the α4(+)/(-)α4 agonist binding site produces increased entry into a common “flip” state would be expected to produce the observed phenomenon of increased bursting at a higher ACh concentration, while also maintaining similar ratios of high- to low-conductance individual openings across a range of ACh concentrations. However, this mechanism is refuted since it predicts that returning from either open state to the common “flip” state within a burst would be followed by a fixed probability of next moving to either of the two observed open-conductance states. In turn, this would be expected frequently to produce bursts typically containing mixtures of low- and high-conductance individual openings. C) A mechanism containing two distinct flip states, each of which is linked to only one of the observed low- or high-conductance open states is compatible with the highly-correlated conductance states of individual openings observed within bursts of LS-isoform α4β2-nAChR. In this mechanism, ACh binding to the α4(+)/(-)α4 agonist binding site similarly enhances entry into either “flip” state, which explains the remaining observed features of similar ratios of high- to low-conductance individual openings (and bursts thereof) at higher ACh concentrations.

However, our findings suggest that the situation is more complicated than previously appreciated. Bursting of both small and large amplitude openings of LS (α4β2)_2_α4-nAChR is similarly enhanced at higher ACh concentrations. The potential mechanisms considered in order to explain our observations are summarized in Fig 12. The simplest possibility is that transitions into both conductance states arise from the same intermediate state. However, amplitudes of openings within bursts were highly correlated. That is, initial small amplitude openings were followed in the same burst by one or more small amplitude openings, while large amplitude openings were also followed essentially exclusively by additional large amplitude events in the same burst. Bursts containing both amplitudes (as would be expected if either could arise stochastically from a single intermediate state) were almost never seen. This implies that the low- and high-conductance openings must arise from two separate intermediate states, both of which are stabilized by agonist binding to the α4(+)/(-)α4 subunit interface site. A similar phenomenon of multiple intermediate closed states has been observed in the cases of the muscle-type nAChR [58] and the glycine receptor, which is a member of the same Cys-loop ligand-gated ion-channel receptor superfamily [59]. It is essential to remember that HS (α4β2)_2_β2-nAChR also produce openings with two characteristic durations. This implies that the HS isoform, too, may access two distinct open states, even if these are not as readily identified since they are not associated with distinct unitary amplitudes and conductance states.

As previously discussed, enhanced macroscopic function of LS α4β2-nAChR in the second, low-affinity phase of function primarily arises from enhanced bursting behavior. However, it is important to note that this effect is somewhat counteracted by an accompanying shortening of open durations within bursts (and of the bursts themselves) without any change in the number of openings per burst. The shortened individual open-event durations indicate that ACh binding at the α4(+)/(-)α4 subunit interface may actually destabilize the open state. A similar phenomenon was reported in [38], at least for the larger amplitude openings arising from LS α4β2-nAChR. This observation is the opposite of what would be expected if ACh binding to the α4(+)/(-)α4 site enhanced function by simply stabilizing the open state(s) of LS (α4β2)_2_α4-nAChR. However, activation of LS isoform responses with the α4(+)/(-)α4 subunit interface-selective agonist NS-9238 actually does produce an elongation of these larger amplitude openings [54]. This indicates that a range of effects can arise from agonist engagement of the low-sensitivity α4(+)/(-)α4 interface, a finding that is compatible with the observation of different binding modes of compounds at this site [30, 60].

The extent to which concatenation of nAChR subunits might alter functional properties of the resulting nAChR subtypes has been a longstanding concern. This anxiety is reasonable, since early concatemeric nAChR constructs did show evidence of low expression and/or expression of unexpected assembly products. These early issues were associated with the use of less than fully-pentameric concatemers and/or linkers of sub-optimal lengths or compositions which could break down and release sub-pentameric products. These nAChR fragments can assemble to form unintended, but functional, byproducts [21, 61–63]. However, more-recent use of fully-pentameric nAChR constructs with optimized linkers has repeatedly been shown to authentically reproduce the macroscopic functional properties of natively expressed nAChR, across a range of subtypes [22, 27, 29, 34, 64–69]. The current study extends this validation to the level of single-channel function. For both HS and LS α4β2-nAChR isoforms, single-channel open durations, numbers of conductance states and their associated conductance values, and low propensity to bursting, are all essentially indistinguishable between isoforms expressed from concatenated or non-concatenated subunits. These findings of similarity even at the detailed single-channel level between α4β2-nAChR isoforms expressed from unlinked or concatenated subunits are further confirmed by another recent publication [54]. We did note some differences in closed-time parameters between the two different expression methods. However, these are difficult to interpret since there is no guarantee that mean numbers of receptors per patch will be comparable between otherwise-identical nAChR expressed from unlinked *vs*. linked subunits. Another potential concern is that even fully-pentameric concatenated nAChR constructs may in theory assemble in two different directions, producing populations with two alternative sets of subunit arrangements [70]. Again, the single-channel properties measured for α4β2-nAChR isoforms assembled using either individual or concatenated subunits were extremely similar. This argues that the concatemeric constructs used in this study authentically reproduced the subunit assemblies adopted in HS and LS α4β2-nAChR isoforms assembled from individual subunits. The experiments using the α4p5W182A mutation provide an even more compelling refutation of this possible confound. Assembly of LSP α4β2-nAChR in the opposite direction to that anticipated would move the mutation outside of the intended α4(+)/(-)α4 subunit interface and produce divergent outcomes between nAChR assembled in the differing directions. However, uniform effects on single-channel properties of LSP α4β2-nAChR were seen. Overall, our data show that well-designed nAChR concatemeric constructs are an excellent model for study of even detailed functional nAChR properties.

To summarize the major findings of this study: We have thoroughly compared the functional properties of HS- and LS-isoform α4β2-nAChR assembled from unlinked subunits to those expressed using fully-linked pentameric constructs. Even at the level of single-channel properties, functional parameters are very similar between receptors expressed with either approach, demonstrating that well-designed concatenated constructs can serve as valid and valuable research models. Using both of these approaches, we have demonstrated that HS-isoform α4β2-nAChR openings are associated with a single low conductance, while LS-isoform α4β2-nAChR produce openings with two distinct (low and high) conductance states. Importantly, both of the conductance states associated with the LS-isoform differ significantly from the single state associated with openings of the HS-isoform. It is well-established that LS-isoform α4β2-nAChR exhibit a second, larger, phase of macroscopic function when exposed to ACh concentrations sufficient to engage a lower-affinity agonist site which only this isoform possesses. The current study indicates that increased macroscopic function is produced by enhanced burst activity. Perhaps surprisingly, increased LS-phase macroscopic function is not associated with a higher prevalence of large-conductance openings. Instead, bursts are observed to contain either low- or high-conductance openings (not mixtures of each), and the prevalence of both types of bursts is similarly increased in the presence of a high ACh concentration. The segregation of the two conductance states into distinct kinds of bursts indicates each mode of LS-isoform α4β2-nAChR may arise from a separate intermediate-closed (“flip”) state, and that higher ACh concentrations serve to stabilize each flip state approximately equally. Finally, the central role of the binding pocket at the α4(+)/(-)α4 subunit interface was confirmed by analysis of LS-isoform α4β2-nAChR harboring a mutation (p5[W182A]) that has previously been shown to reduce the LS-phase of the macroscopic concentration/response curve [27]. As would be predicted, this mutation reduced or abolished the just-mentioned changes in single-channel behaviors produced by a high ACh concentration at non-mutated LS-isoform α4β2-nAChR. These findings of the current study may have substantial practical effects. Most directly, this work provides novel functional insights that may supply useful guidance for drug design and development to differentially affect the function of HS and LS α4β2-nAChR isoforms. In particular, the finding that interactions of different compounds at the α4(+)/(-)α4 binding site of LS α4β2-nAChR (ACh in this study, NS-9238 in [54]) may result in dissimilar functional effects suggests the possibility to fine-tune drug effects on LS isoform efficacy. This may be critically important; α4β2*-nAChR are the intended target of varenicline, the most successful smoking cessation pharmacotherapy currently available [14, 15], and several studies indicate that altering the balance of α4β2-nAChR isoform function can produce physiologically significant effects [19, 20, 71–73]. In addition, improved opportunities to identify and distinguish between the isoforms of α4β2-nAChR will enable improved knowledge of their contributions, more generally, in normal physiology and disease states. This would address a significant gap in our understanding–that of the precise physiological roles of HS and LS α4β2-nAChR isoforms.

## Supporting information

S1 Data Calculated values for closed, open, amplitude, and event frequency data for examined constructs. Excel file contains single-channel data for unlinked and concatenated single-channel parameters calculated using QuB for each patch. Averages, S.E.M, and outlier test calculations are included.
(XLSX)

S2 Data Amplitude values measured at transmembrane potentials from -70 to -140 mV. Excel files contain single-channel amplitudes as determined using QuB for each construct at the different transmembrane potentials. For LS and LSP α4β2-nAChR data is separated by small and large amplitude events.
(XLSX)

S3 Data Interburst duration raw data values. Provided data consists of the raw interburst durations as determined using QuB. Data for each patch has been pooled into a single column. LS, LSP, and α5p5W182A α4β2-nAChR construct data is separated by event amplitude.
(XLSX)

S4 Data Intraburst interval raw data values. Provided data consists of raw intraburst interval raw data values as determined using QuB for each patch. LS, LSP, and α5p5W182A construct data is separated by event amplitude.
(XLSX)

S5 Data The number of events evoked within a burst for LS α4β2-nAChR using the low and high ACh concentrations. Raw data were determined using QuB, and LS α4β2-nAChR data is separated by amplitude designation.
(XLSX)

S6 Data Average burst Popen values stimulated by the low and high ACh concentrations for the LS α4β2-nAChR isoform. Values were determined using QuB, and LS α4β2-nAChR data is separated by amplitude designation.
(XLSX)

S7 Data Burst duration values for the unlinked constructs. Values were determined using QuB, and LS α4β2-nAChR data is separated by amplitude designation.
(XLSX)

## Abbreviations

ACh: acetylcholine
HS: high-sensitivity
HSP: high-sensitivity concatenated pentamer
LL: log likelihood
LS: low-sensitivity
LSP: low-sensitivity concatenated pentamer
LSP-α4p5W182A: tryptophan (W) to alanine (A) mutation at amino acid 182 of only the position-five α4 subunit within the low-sensitivity concatenated pentamer;
MIL: maximum interval likelihood
O_L_: large amplitude openings
OR2: oocyte Ringer’s solution
O_S_: small amplitude openings
P_open_: open probability within a burst
SKM: segmental K-means
T_crit_: maximum interburst closed duration
